# Impact of Physical Activity on Stroke and Mortality in Individuals With and Without Atrial Fibrillation: The HUNT Study and the Tromsø Study

**DOI:** 10.1161/JAHA.126.048812

**Published:** 2026-08-05

**Authors:** Kristoffer Robin Johansen, Bjarne Martens Nes, Vegard Malmo, Marius Myrstad, Dag S Thelle, Kim Arne Heitmann, Ellisiv Bøgeberg Mathiesen, Anne Elise Eggen, Tom Wilsgaard, Maja‐Lisa Løchen, Bente Morseth, the Norwegian Exercise and Atrial Fibrillation Initiative Investigators

**Affiliations:** ^1^ School of Sport Sciences, Faculty of Health Sciences UiT The Arctic University of Norway Tromsø Norway; ^2^ Department of Cardiology University Hospital of North Norway Tromsø Norway; ^3^ Department of Circulation and Medical Imaging Norwegian University of Science and Technology, NTNU Trondheim Norway; ^4^ Clinic of Cardiology, St. Olavs Hospital Trondheim University Hospital Trondheim Norway; ^5^ Department of Internal Medicine Bærum Hospital, Vestre Viken Hospital Trust N‐1346 Gjettum Norway; ^6^ Department of Biostatistics, Institute of Basic Medical Sciences University of Oslo Oslo PO Box 1122 Blindern Norway; ^7^ School of Public Health and Community Medicine, Institute of Medicine Sahlgrenska Academy at University of Gothenburg Göteborg Po Box 414, SE404 30 Sweden; ^8^ Department of Clinical Medicine UiT The Arctic University of Norway Tromsø Norway; ^9^ Department of Neurology University Hospital of North Norway Tromsø Norway; ^10^ Department of Community Medicine, Faculty of Health Sciences UiT The Arctic University of Norway Tromsø Norway

**Keywords:** atrial fibrillation, mortality, physical activity, stroke, the HUNT study, the Tromsø study, Epidemiology, Exercise, Cerebrovascular Disease/Stroke, Atrial Fibrillation

## Abstract

**Background:**

Although physical activity (PA) is associated with a reduced risk of stroke and mortality, its impact among individuals with atrial fibrillation (AF) remains insufficiently explored. Therefore, the aim of this study was to examine the impact of PA on stroke and mortality, both overall and with respect to AF diagnosis.

**Methods:**

We included 87 340 participants, of which 6539 had AF, from 2 Norwegian population‐based studies, the Tromsø Study and the HUNT (Trøndelag Health Study). Information about AF, stroke, and death were obtained from national health registries. Participants were classified as inactive (reference) or performing low, moderate, or high PA on the basis of a questionnaire. The data were analyzed using multivariable adjusted flexible parametric survival models.

**Results:**

During a median follow‐up of 13.5 years, 3415 strokes and 7833 deaths occurred. The respective stroke risk reduction associated with low, moderate, and high PA was 9% (95% CI, 1%–16%), 19% (95% CI, 11%–27%) and 18% (95% CI, 8%–26%). Mortality was reduced by 11% (95% CI, 7%–14%), 18% (95% CI, 14%–22%), and 22% (95% CI, 18%–27%). Similar benefits were observed for both stroke and mortality when stratifying by presence or absence of AF. Among individuals with AF, an increased life expectancy was observed, with gains of 0.50 (95% CI, 0.22–0.78), 0.66 (95% CI, 0.31–1.01) and 1.15 (95% CI, 0.77–1.53) years, respectively.

**Conclusions:**

PA was associated with a reduced risk of stroke and mortality, and similar benefits were observed irrespective of AF diagnosis. Our findings suggest that PA may complement current medical treatment strategies in individuals with AF.

## Graphical abstract

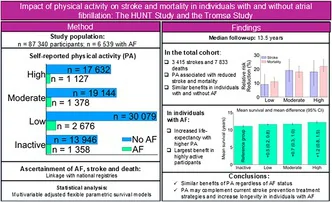

**Nonstandard Abbreviations and Acronyms**
HUNT: Trøndelag Health Study
NPR: Norwegian Patient Registry
PA: physical activity
PH: proportional hazards

**Clinical Perspective**

**What Is New?**

- Physical activity was associated with a similar stroke and mortality risk reduction in individuals with and without atrial fibrillation.
- Even low levels of physical activity were associated with a reduced rate of stroke and increased life expectancy in individuals with atrial fibrillation, although the greatest benefit was observed in the most active individuals.

**What Are the Clinical Implications?**

- These findings suggest that physical activity may complement current stroke treatment strategies among individuals with atrial fibrillation.

Atrial fibrillation (AF) is the most common arrhythmia worldwide.^1^ The estimated lifetime risk of AF from midlife is ≈20% to 35% depending on risk factors,^2^, ^3^, ^4^ and the prevalence of AF is expected to double by 2060.^5^ AF is associated with multiple cardiovascular conditions and a reduced life expectancy.^6^ Notably, stroke rates may be >5 times higher in individuals with AF, depending on concomitant risk factors.^7^, ^8^ Consequently, ≈1 in 5 ischemic strokes in Western Europe, North America, and Australia are attributable to AF.^9^ Management of cardiovascular comorbidities and prevention of adverse outcomes such as stroke and mortality are pillars of modern AF care.^10^ Physical inactivity often coexists with other cardiovascular risk factors, and increasing the level of physical activity (PA) may benefit patients with AF both in terms of better risk factor management and beneficial effects on cardiovascular adverse outcomes.

PA is highlighted by the European Society of Cardiology and the American Heart Association as an essential measure in the primary prevention of stroke^11^, ^12^ and is furthermore associated with a reduced risk of cardiovascular and all‐cause death.^13^ PA may therefore serve as a promising lifestyle intervention both in primary prevention of AF^14^, ^15^, ^16^ and in secondary prevention of stroke and mortality in individuals with AF. However, the impact of PA on the risk of stroke and mortality, which may be increased in individuals with AF, remains insufficiently investigated. Previous studies addressing the impact of PA on stroke risk report inconclusive results, whereas associations between PA and mortality have been inconsistent.^17^, ^18^, ^19^, ^20^ However, constraints related to sample size or follow‐up duration may have limited existing studies, thereby warranting further investigation.

The purpose of this study was to examine the prospective association between PA and the risk of stroke and mortality, both overall and with respect to AF diagnosis, in a large cohort based on 2 population‐based studies in Norway.

## Methods

### Data Availability

#### The Trøndelag Health Study

The HUNT (Trøndelag Health Study) has invited people aged 13 to 100 years to 4 surveys between 1984 and 2019. Comprehensive data from >140 000 people having participated at least once and biological material from 78 000 people are collected. The data are stored in HUNT databank and biological material in HUNT biobank. HUNT Research Centre has permission from the Norwegian Data Inspectorate to store and handle these data. The key identification in the database is the personal identification number given to all Norwegians at birth or immigration, while deidentified data are sent to researchers upon approval of a research protocol by the Regional Ethical Committee and HUNT Research Centre. To protect participants' privacy, HUNT Research Centre aims to limit storage of data outside HUNT databank and cannot deposit data in open repositories. HUNT databank has precise information on all data exported to different projects and is able to reproduce these on request. There are no restrictions on data export given approval of application to HUNT Research Centre. For more information, see http://www.ntnu.edu/hunt/data.

#### The Tromsø Study

The data underlying this article cannot be made publicly available due to General Data Protection Regulation. The legal restrictions on data availability are set by the Tromsø Study Data and Publication Committee to control for data sharing, including publication of data sets with the potential of reverse identification of deidentified sensitive participant information. However, the data can be made available on application to the Tromsø Study Data and Publication Committee (The Tromsø Study, Department of Community Medicine, Faculty of Health Sciences, UiT The Arctic University of Norway; Email: tromsous@uit.no).

### Design and Study Population

This prospective study includes a cohort of 87 340 individuals from 2 large Norwegian population‐based studies, the Tromsø Study^21^ and the HUNT Study^22^ (Figure 1).

**Figure 1:**
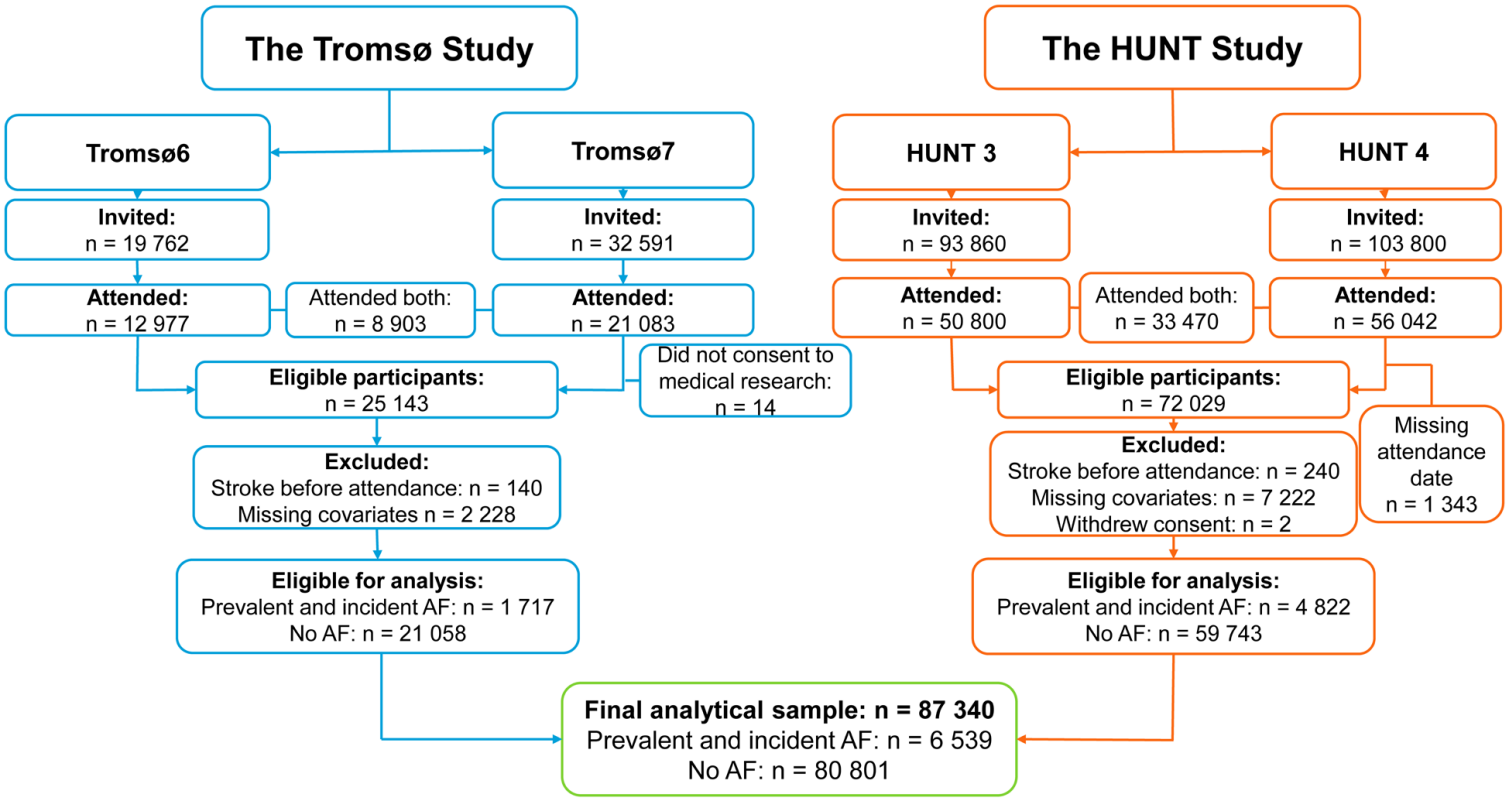
Flowchart with an overview of participants included. Participants in the study were defined as individuals who responded to the main questionnaire and provided information about their date of participation.

From the Tromsø Study, we included participants from the sixth and seventh surveys (Tromsø6, Tromsø7), conducted in 2007 to 2008 and 2015 to 2016, respectively.^23^, ^24^ The Tromsø Study includes residents from the municipality of Tromsø. Among those invited to Tromsø6, 12 977 (66%) participants aged 30 to 87 years attended, while in Tromsø7, 21 083 (65%) participants aged 40 to 99 years attended. Upon invitation, all participants received a questionnaire and shortly thereafter attended a clinical examination. A more detailed description is given elsewhere.^23^, ^24^

From the HUNT Study, we included participants attending the third wave in 2006 to 2008 and the fourth wave in 2017 to 2019. The HUNT participants received a questionnaire upon invitation and subsequently underwent a clinical examination.^22^, ^25^ Eligible participants were inhabitants of Nord‐Trøndelag County aged ≥20 years, and in total 50 800 (54.1%) participants attended HUNT3 and 56 042 (54.0%) attended HUNT4.^22^

### Classification of AF

To study the impact of AF on the association between PA and stroke and mortality, we classified prevalent and incident AF cases. Diagnoses for classification of AF were retrieved from several registries: (1) The diagnosis registry of the University Hospital of North Norway (available for Tromsø6 and Tromsø7 participants), (2) The diagnosis registry of the 2 hospitals in Nord‐Trøndelag County (available for HUNT3 participants), and (3) the NPR (Norwegian Patient Registry). The diagnosis registries provide diagnoses that were confirmed by a physician's review of medical journals and a 12‐lead ECG,^26^, ^27^ while the NPR is an administrative health registry that classified AF with *International Classification of Diseases*, *Tenth Revision* (*ICD‐10*) codes I48 to I48.9.^28^ Participants were classified with AF (n=6539 in the final analytical sample) according to hospital‐confirmed diagnoses (58.9%; n=3850). However, for the HUNT4 cohort, hospital diagnoses were not available, and to supplement the registry at the University Hospital of North Norway beyond 2016, we used *ICD* codes from NPR for these subcohorts (available until the end of 2021) (41.1%; n=2689).

In the Tromsø Study, participants were linked to the diagnosis registry at the University Hospital of North Norway (including attendance at the outpatient clinic) through their 11‐digit identification number, which is the only hospital in this region, and to the Causes of Death Registry at the National Institute of Public Health.^26^ To identify AF, a broad search using the *ICD*, *Ninth Revision* (*ICD‐9*) codes 410 to 414, 427, 428, 430 to 438, and 798 to 799 and the *ICD‐10* codes I20 to I25, I46 to I48, I50, I60 to I69, R96, R98, and R99, and specific search terms such as *arrhythmia*, *tachycardia*, *palpitations*, *atrial flutter*, and *AF* was conducted.^29^ Following a detailed protocol and using medical hospital records, an independent endpoint committee validated all possible events that were identified through the search.^26^, ^29^ AF cases were confirmed on an ECG. AF occurring during the last 7 days of life and AF cases within 28 days after acute myocardial infarction or in relation to cardiac surgery were not classified as AF cases.^29^

A similar procedure was conducted in HUNT. Specifically, the participants' identification number was linked with the registries of the 2 hospitals in the region.^27^ *ICD‐9* code 427.3 and *ICD‐10* codes I48.0 to I48.9 for AF and atrial flutter were used to identify potential AF cases.^27^ AF was verified by a cardiologist on the basis of either an ECG that identified AF or atrial flutter using standard criteria,^30^ or if an ECG was not available, the medical record was reviewed for interpretation.^27^

An exact AF diagnosis date was not provided by the NPR (diagnosis month provided) or from the diagnosis registry applied in HUNT3 (diagnosis year provided). Therefore, date of diagnosis was set to the middle date of the month (NPR) or year (HUNT3) for these registries. Because of this, some participants had the date of AF occurring after the date of death (n=67). This was mitigated by changing the date of AF according to NPR diagnoses instead when available (n=66) or by changing the date of AF to the same day as the date of death (n=1).

All participants with prevalent and incident AF were followed from the first survey that they attended. To do this, we applied a multiple records approach. Participants with incident AF had 2 rows in the data file and were classified as not having AF from date of attendance and until the date of AF. From date of AF, they were reclassified as having AF and followed until stroke, death, or end of follow‐up, whichever came first.

### Ascertainment of Outcomes and Follow‐Up

The outcomes of interest were ascertained by linking participants' data in the Tromsø Study and HUNT with data on stroke and mortality from the NPR.^28^ Participants were followed from date of attendance and until the event of interest occurred, a competing event (death without stroke in the analysis of stroke), or until end of follow‐up (December 31, 2021). *ICD‐10* codes I61, I63, and I64 were used to classify stroke. Associations with subtypes (intracerebral hemorrhage: I61; ischemic stroke: I63 and I64) were also explored in supplemental analyses. For the analyses of stroke and mortality, all strokes that occurred before start of follow‐up were excluded (n=380). Furthermore, in the analysis of the outcome mortality, any stroke that occurred before or on the same date as AF (n=468) was viewed as a confounding factor. Consequently, when this occurred, participants were censored on the date of AF instead of being reclassified.

### Exposure

The main exposure was self‐reported PA at the first study attendance, obtained with the frequency–intensity–duration questionnaire.^31^ Frequency of PA was assessed with the following alternatives: “never” [0], “less than once a week” [0.5], “once a week” [1], “2 to 3 times per week” [2.5], or “almost every day” [5]. Duration of exercise was assessed using the alternatives “less than 15 minutes” [7.5], “15 to 29 minutes” [22], “30 minutes to 1 hour” [45], or “more than 1 hour” [60]. Exercise intensity was assessed as either “I take it easy without breaking into a sweat or losing my breath” [3], “I push myself so hard that I lose my breath and break into a sweat” [6], or “I push myself to near exhaustion” [9]. Based on the numbers in brackets, we created a PA index to reflect minutes of metabolic equivalents of task per week (MET min/wk), which is the product of intensity, minutes of exercise, and frequency of exercise as previously described.^32^ Furthermore, we categorized participants according to their PA level as either “inactive” (0 MET min/wk), “low” (1–449 MET min/wk), “moderate” (450–899 MET min/wk), or “high” (≥900 MET min/wk). For clarity, further elaboration with example calculation is given in Table S1.

To conduct a subgroup analysis to evaluate agreement between self‐reported PA and triaxial accelerometery, we identified a subgroup who attended Tromsø7 with device‐measured PA.^24^ In total, 6778 participants from Tromsø7 were invited to wear an accelerometer (ActiGraph wGT3X‐BT, Actigraph, LLC, Pensacola, FL), of whom 6 125 had valid accelerometer data.^33^ In this study, 5 605 (n=495 with AF) had available accelerometer data.

### Covariates

Height and weight were measured with the participants wearing light clothing without shoes. Resting heart rate and blood pressure readings were recorded in a seated position with 1‐minute intervals 3 times after a 2‐minute seated rest with a Dinamap device (Tromsø6 and Tromsø7: ProCare 300; HUNT3: 845XT Critikon; HUNT4: CARESCAPE V100).^22^, ^23^, ^24^, ^25^ The average of the 2 last measurements was used. Blood samples were collected in a nonfasted state in both studies. Smoking (“never smoker,” “previous smoker,” “current smoker”), frequency of alcohol consumption (“not in the past year,” “monthly or less,” “2 to 4 times per month,” “‘2 to 3 times per week,” “4 or more times per week”), and university education (yes/no) were self‐reported (Tables S2–S4). Coronary heart disease was defined by 2 distinct questions: “Do you have or have you had angina/heart attack?” categorized as yes or no. Prevalent diabetes and heart failure (the latter not available in Tromsø6), history of stroke, and use of blood pressure medication (never or currently/previously) were self‐reported. We further calculated a nonsex CHA_2_DS_2_‐VA^10^ score based on age and self‐reports of heart failure, use of blood pressure medication, history of stroke and coronary heart disease, and diabetes (Table S5). Based on this, we calculated the CHA_2_DS_2_‐VA score and defined 3 categories of stroke risk (0=low risk, 1=intermediate risk, ≥2=high risk).

Information on anticoagulant (Anatomical Therapeutic Chemical Classification System: B01AA03, B01AF02, B01AE07, B01AF01) and antiplatelet (Anatomical Therapeutic Chemical Classification System: B01AC04, B01AC06, B01AC07, B01AC24, B01AC22) use was available only in individuals who attended the Tromsø Study. The participants in Tromsø6 and Tromsø7 reported all medications used regularly the last 4 weeks preceding examination, which was then coded according to the Anatomical Therapeutic Chemical Classification System version 2016.^34^ Information about dosage was not available.

### Statistical Analysis

All statistical analyses were performed with Stata version 18 (StataCorp, College Station, TX). We report descriptive characteristics as mean±SD for continuous variables (unless otherwise stated), and as number and percentage for categorical variables. All main analyses were multivariable adjusted for age, sex, education, body mass index, smoking status, frequency of alcohol consumption, coronary heart disease, and study cohort, with inactive participants as the reference group. Our assumptions of the causal relationship between PA and the outcomes of interest are presented in a directed acyclic graph (Figure S1) according to current recommendations.^35^ We conducted analyses in the overall cohort and in subcohorts with and without AF. In addition, effect modification by AF was tested by comparing an interaction model that included AF as a main effect and the cross‐product term [PA×AF] against a model that included only AF as a main effect. The interaction was assessed with a likelihood ratio test.

We initially applied a Cox proportional hazards (PH) model to analyze the data. To assess the PH assumption, we inspected log‐minus‐log plots as well as conducting tests of Schoenfeld's residuals (Tables S6 and S7; Figures S2 and S3). Schoenfeld's test indicated a violation of the PH assumption for the outcome mortality (*P*<0.05). This was further corroborated in an analysis with a time‐dependent Cox model where an interaction between PA and follow‐up time was detected (*P*<0.05). Therefore, further analyses were conducted with flexible parametric models to accommodate the time‐dependent effects of PA.^36^ A flexible PH model for the outcome stroke was applied, as the PH assumption was deemed appropriate for this outcome. Number of knots used to parameterize the baseline hazard, and the time‐dependent effect of PA was assessed with Akaike's information criterion.

We estimated absolute risks of PA associated with the outcomes of interest. For the outcome mortality, the standardized failure function was estimated.^37^ However, for the outcome stroke, we needed to control for a competing mortality risk.^38^ Therefore, the cause‐specific cumulative incidence function was estimated.^38^ To do this, we modeled the stroke rate and the competing mortality rate with 2 separate models. Subsequently, regression standardization was conducted to construct the standardized cause‐specific cumulative incidence function.^39^ The absolute risk, risk ratio, risk difference, and corresponding 95% CI after 15 years (the maximum follow‐up time rounded to the nearest integer) is derived from these curves and presented.

Because the impact of PA on the cause‐specific stroke rate may be of great importance,^38^, ^40^ we also present hazard ratio (HR) plots (time‐dependent when appropriate). Moreover, to obtain an estimate of the life expectancy in participants with AF during the study period, we estimated the restricted mean survival time and the corresponding difference^41^ for the outcome mortality.

### Sensitivity Analyses

We conducted 6 specific sensitivity analyses in individuals with AF:

1. To evaluate the potential for unmeasured confounding by anticoagulation treatment, we reanalyzed the association between PA and stroke in individuals with AF who participated in the Tromsø Study (n=1 717) after further adjustment for anticoagulants.
2. In the analysis of stroke, we additionally adjusted for the CHA_2_DS_2_‐VA score and components of the score to evaluate the potential for residual confounding.
3. We performed subgroup analyses in participants categorized as being at low, intermediate, and high risk of stroke according to the CHA_2_DS_2_‐VA score. Due to a lack of statistical power, this subgroup analysis was conducted with stroke and mortality as a composite outcome.
4. Individuals with incident AF may cause bias to our estimates because their PA levels are ascertained before they are diagnosed with AF. Therefore, we censored these individuals on the date of AF and reanalyzed the data.
5. Furthermore, since prevalent AF cases are followed from the date of attendance instead of the time of diagnosis, the number of person‐years observed in this subgroup is possibly underestimated. It is also possible that these individuals may constitute a somewhat selected group of individuals with AF who are relatively healthy. Thus, we also reanalyzed the data after excluding these participants.
6. Because 17.2% of the participants with AF had missing data on covariates that entered the regression model, we modeled the cause‐specific stroke rate after performing multivariate imputation with chained equations.

We performed 4 additional sensitivity analyses in the total cohort:

1. We excluded participants with <2 years of follow‐up to examine the potential effect of reverse causation for the outcome stroke.
2. Because it may be unclear whether cardiovascular disease is a mediator or confounder, we excluded participants self‐reporting coronary heart disease.
3. We present descriptive statistics and Spearman's correlation coefficients to examine agreement between self‐reported PA and device‐measured PA.
4. We examined whether the association between PA and the outcomes of interest was modified by age and sex.

### Ethics

The present study was approved by the Regional Committee for Medical and Health Research Ethics (REK: 2020–175 586) and the data protection officer at UiT The Arctic University of Norway in collaboration with the Norwegian Agency for Shared Services in Education and Research. All participants provided written informed consent to participate.

## Results

The mean age at baseline was 50.9±15.1 years, and 47.4% were men. Descriptive statistics by level of PA are given in Table 1. Participants with AF were older, had a lower proportion of women, and a higher proportion of comorbidity than those without AF (Tables S8 and S9). In total, 15 304 (17.5%) were classified as inactive, while 32 755 (37.5%), 20 522 (23.5%), and 18 759 (21.5%) were classified as performing low, moderate, and high amounts of PA, respectively. Participants reporting higher levels of PA were more educated, smoked less, had a lower body mass index and a lower prevalence of cardiovascular risk factors, and were at lower risk of stroke according to the CHA_2_DS_2_‐VA score.

**Table 1:** Descriptive Characteristics of the Total Population According to PA Level

	Inactive (n=15 304)	Low (n=32 755)	Moderate (n=20 522)	High (n=18 759)
Age, y	50.8±16.3	52.4±14.9	50.9±14.3	48.5±14.9
Female sex	6 484 (42.4)	17 743 (54.2)	12 169 (59.3)	9 548 (50.9)
Male sex	8 820 (57.6)	15 012 (45.8)	8 353 (40.7)	9 211 (49.1)
Atrial fibrillation*	1 358 (8.9)	2 676 (8.2)	1 378 (6.7)	1 127 (6.0)
MET min/wk	0.0±0.0	221.7±109.7	658.9±55.5	1 285.6±411.2
Height, cm	172.1±9.5	170.7±9.3	170.9±9.0	172.3±9.1
Weight, kg	83.2±17.4	80.1±16.0	78.3±15.0	77.9±14.7
Body mass index, kg/m^2^	28.0±5.1	27.4±4.6	26.7±4.3	26.2±4.0
University educated	3 929 (25.7)	11 585 (35.4)	9 227 (45.0)	8 403 (44.8)
Smoking status				
Never	5 277 (34.5)	13 419 (41.0)	9 396 (45.8)	9 180 (48.9)
Previously	5 148 (33.6)	12 157 (37.1)	8 001 (39.0)	7 197 (38.4)
Currently	4 879 (31.9)	7 179 (21.9)	3 125 (15.2)	2 382 (12.7)
Frequency of alcohol consumption				
Not in the past year	1 667 (10.9)	3 094 (9.4)	1 408 (6.9)	1 152 (6.1)
Monthly or less	5 429 (35.5)	10 145 (31.0)	5 585 (27.2)	5 237 (27.9)
2–4 times/mo	6 120 (40.0)	13 840 (42.3)	9 107 (44.4)	8 242 (43.9)
2–3 times/wk	1 675 (10.9)	4 661 (14.2)	3 719 (18.1)	3 417 (18.2)
≥4 times/wk	413 (2.7)	1 015 (3.1)	703 (3.4)	711 (3.8)
Systolic blood pressure, mm Hg	130.0±18.6	129.8±19.3	127.6±18.8	127.0±18.2
Diastolic blood pressure, mm Hg	74.2±11.2	74.5±11.0	73.6±10.7	73.1±10.7
Resting heart rate, beats/min	72.8±12.1	70.6±11.7	68.6±11.4	66.7±11.4
Total cholesterol, mmol/L	5.4±1.1	5.5±1.1	5.4±1.1	5.3±1.1
High‐density lipoprotein, mmol/L	1.3±0.3	1.4±0.4	1.4±0.4	1.5±0.4
Triglycerides, mmol/L	1.8±1.2	1.7±1.0	1.5±0.9	1.4±0.9
Blood pressure medication	3 162 (24.4)	6 819 (23.5)	3 511 (19.7)	2 626 (16.7)
Anticoagulants†	49 (3.5)	179 (1.8)	73 (1.3)	61 (1.1)
Antiplatelets†	205 (14.5)	872 (8.6)	402 (7.0)	287 (5.2)
Coronary heart disease	997 (6.5)	1 813 (5.5)	863 (4.2)	677 (3.6)
Heart failure‡	201 (1.4)	265 (1.0)	126 (0.7)	97 (0.6)
Diabetes	788 (5.2)	1 508 (4.6)	723 (3.5)	520 (2.8)
CHA_2_DS_2_‐VA score‡				
Low risk	6 075 (49.8)	12 021 (50.3)	8 608 (56.0)	8 042 (58.6)
Intermediate risk	3 218 (26.4)	6 277 (26.2)	3 883 (25.2)	3 407 (24.8)
High risk	2 906 (23.8)	5 619 (23.5)	2 894 (18.8)	2 268 (16.5)

Descriptive characteristics according to PA level. Values are presented as mean±SD for continuous variables and n (%) for categorical variables. MET indicates metabolic equivalent of task; and PA, physical activity.

* Prevalent and incident atrial fibrillation cases.

^†^ Information only available in the Tromsø Study.

^‡^ Information on heart failure not available in the Tromsø6 Study cohort.

### PA and Risk of Incident Stroke

During a median follow‐up of 13.5 years, 3 415 strokes occurred, of which 87.0% (n=2 970) were ischemic, while 13.0% (n=445) were classified as intracerebral hemorrhage. The absolute risk of stroke after 15 years was 4.6% (95% CI, 4.4%–4.8%) in individuals without AF and 16.7% (95% CI, 15.5%–17.9%) in individuals with AF.

Stroke and mortality event rates, absolute risks, risk ratios, and risk differences for the total cohort and for individuals with and without AF are presented in Table 2. In the total cohort, higher PA levels were associated with a 9% to 18% reduced risk of stroke compared with inactive individuals. Similar effects were observed when stratifying by AF diagnosis (*P* interaction=0.102), with a 12% to 20% risk reduction observed in individuals with AF. The cause specific cumulative incidence for stroke according to PA level in individuals with AF is presented in Figure 2.

**Table 2:** Stroke and Mortality Event Rates, Absolute Risks, Risk Ratios and Risk Differences for the Total Cohort and for Individuals With and Without AF

	Events, n (%)	Rate per 100 000 (95% CI)	Absolute risk (95% CI)	Risk ratio (95% CI)	Risk difference (95% CI)
Total sample					
Stroke					
Inactive	762 (5.0)	487 (454 to 523)	0.060 (0.056 to 0.064)	1 Reference	1 Reference
Low	1 462 (4.5)	437 (416 to 460)	0.055 (0.052 to 0.058)	0.91 (0.84 to 0.99)	−0.005 (−0.010 to 0.000)
Moderate	664 (3.2)	321 (297 to 346)	0.048 (0.045 to 0.052)	0.81 (0.73 to 0.89)	−0.012 (−0.017 to −0.006)
High	527 (2.8)	290 (267 to 316)	0.049 (0.045 to 0.054)	0.82 (0.74 to 0.92)	−0.010 (−0.016 to −0.005)
All‐cause death					
Inactive	1 991 (13.0)	1 249 (1 195 to 1 305)	0.147 (0.142 to 0.153)	1 Reference	1 Reference
Low	3 353 (10.2)	985 (952 to 1 019)	0.131 (0.127 to 0.135)	0.89 (0.86 to 0.93)	−0.016 (−0.022 to −0.010)
Moderate	1 475 (7.2)	704 (669 to 740)	0.120 (0.115 to 0.125)	0.82 (0.78 to 0.86)	−0.027 (−0.034 to −0.020)
High	1 014 (5.4)	552 (519 to 587)	0.114 (0.109 to 0.120)	0.78 (0.73 to 0.82)	−0.033 (−0.040 to −0.025)
Individuals without AF					
Stroke					
Inactive	592 (4.2)	397 (367 to 431)	0.051 (0.047 to 0.055)	1 Reference	1 Reference
Low	1 193 (4.0)	374 (353 to 396)	0.048 (0.045 to 0.051)	0.95 (0.86 to 1.04)	−0.003 (−0.007 to 0.002)
Moderate	524 (2.7)	263 (242 to 287)	0.041 (0.037 to 0.044)	0.80 (0.71 to 0.90)	−0.010 (−0.015 to −0.005)
High	433 (2.5)	248 (226 to 272)	0.043 (0.039 to 0.047)	0.84 (0.74 to 0.95)	−0.008 (−0.014 to −0.003)
All‐cause death					
Inactive	1 372 (9.8)	905 (859 to 955)	0.113 (0.108 to 0.119)	1 Reference	1 Reference
Low	2 397 (8.0)	740 (710 to 770)	0.101 (0.097 to 0.105)	0.90 (0.85 to 0.95)	−0.012 (−0.018 to −0.006)
Moderate	1 071 (5.6)	532 (501 to 565)	0.092 (0.087 to 0.097)	0.81 (0.76 to 0.87)	−0.021 (−0.028 to −0.014)
High	760 (4.3)	430 (401 to 462)	0.089 (0.083 to 0.095)	0.79 (0.73 to 0.85)	−0.024 (−0.032 to −0.017)
Individuals with AF					
Stroke					
Inactive	170 (12.5)	2 299 (1 978 to 2 672)	0.195 (0.170 to 0.225)	1 Reference	1 Reference
Low	269 (10.1)	1 759 (1 561 to 1 982)	0.156 (0.140 to 0.175)	0.80 (0.67 to 0.95)	−0.039 (−0.071 to −0.007)
Moderate	140 (10.2)	1 796 (1 522 to 2 120)	0.171 (0.147 to 0.199)	0.88 (0.71 to 1.07)	−0.024 (−0.062 to 0.013)
High	94 (8.3)	1 401 (1 145 to 1 715)	0.157 (0.130 to 0.189)	0.80 (0.64 to 1.02)	−0.038 (−0.079 to 0.002)
All‐cause death					
Inactive	619 (45.6)	7 818 (7 225 to 8 458)	0.537 (0.514 to 0.561)	1 Reference	1 Reference
Low	956 (35.7)	5 889 (5 528 to 6 275)	0.491 (0.473 to 0.509)	0.91 (0.87 to 0.96)	−0.046 (−0.073 to −0.020)
Moderate	404 (29.3)	4 877 (4 423 to 5 376)	0.472 (0.448 to 0.498)	0.88 (0.82 to 0.94)	−0.065 (−0.098 to −0.032)
High	254 (22.5)	3 633 (3 213 to 4 109)	0.426 (0.397 to 0.457)	0.79 (0.73 to 0.86)	−0.111 (−0.148. −0.073)

Absolute risk, risk ratio, and risk difference are given after 15 years of follow‐up and have been adjusted for age, sex, body mass index, university education, smoking status, frequency of alcohol consumption, coronary heart disease, and study cohort. Participants were classified according to PA level as either inactive (0 MET min/wk) or performing low (1–449 MET min/wk), moderate (450–899 MET min/wk) or high (≥900 MET min/wk) amounts of PA.

AF indicates atrial fibrillation; MET, metabolic equivalent of task; and PA, physical activity.

**Figure 2:**
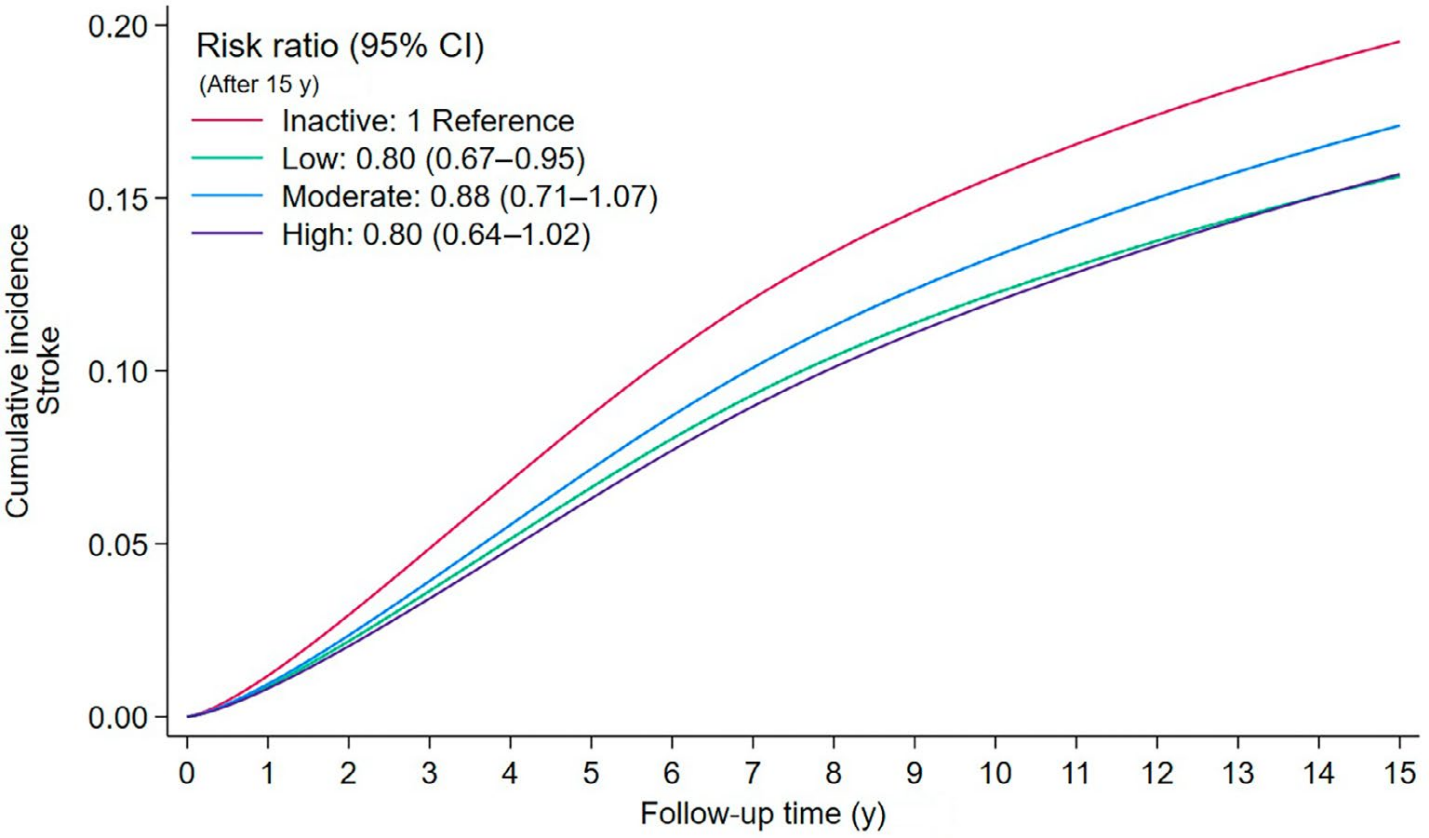
Cause‐specific cumulative incidence function indicating the absolute risk of stroke according to PA level in individuals with AF. Risk ratio and 95% CI are given after 15 years. The rate of stroke and the rate of the competing event were estimated with 2 separate flexible parametric models that were subsequently combined to construct the cumulative incidence function with regression standardization. Both models were adjusted for age, sex, body mass index, university education, smoking status, frequency of alcohol consumption, coronary heart disease, and study cohort. Furthermore, the baseline hazard function for the rate of stroke and the rate of the competing event were modeled with restricted cubic splines with knots placed at the 25th, 50th, and 75th centile of the uncensored log event time. AF indicates atrial fibrillation; and PA, physical activity.

As shown in Figure 3, higher PA levels were associated with a significant reduction in the cause‐specific hazard for stroke, with comparable risk reductions in individuals with and without AF. In highly active individuals, the cause‐specific stroke rate was reduced by 21% (95% CI, 11%–31%) and 32% (95% CI, 11%–48%) in individuals without and with AF, respectively. The difference between the risk ratio derived from the cause‐specific cumulative incidence function after 15 years and the cause‐specific HR among participants with AF might be due to a large competing mortality risk of 38.7% (95% CI, 37.3%–40.1%) and the pronounced competing mortality rate reduction of 39% (95% CI, 28%–49%) associated with high PA.

**Figure 3:**
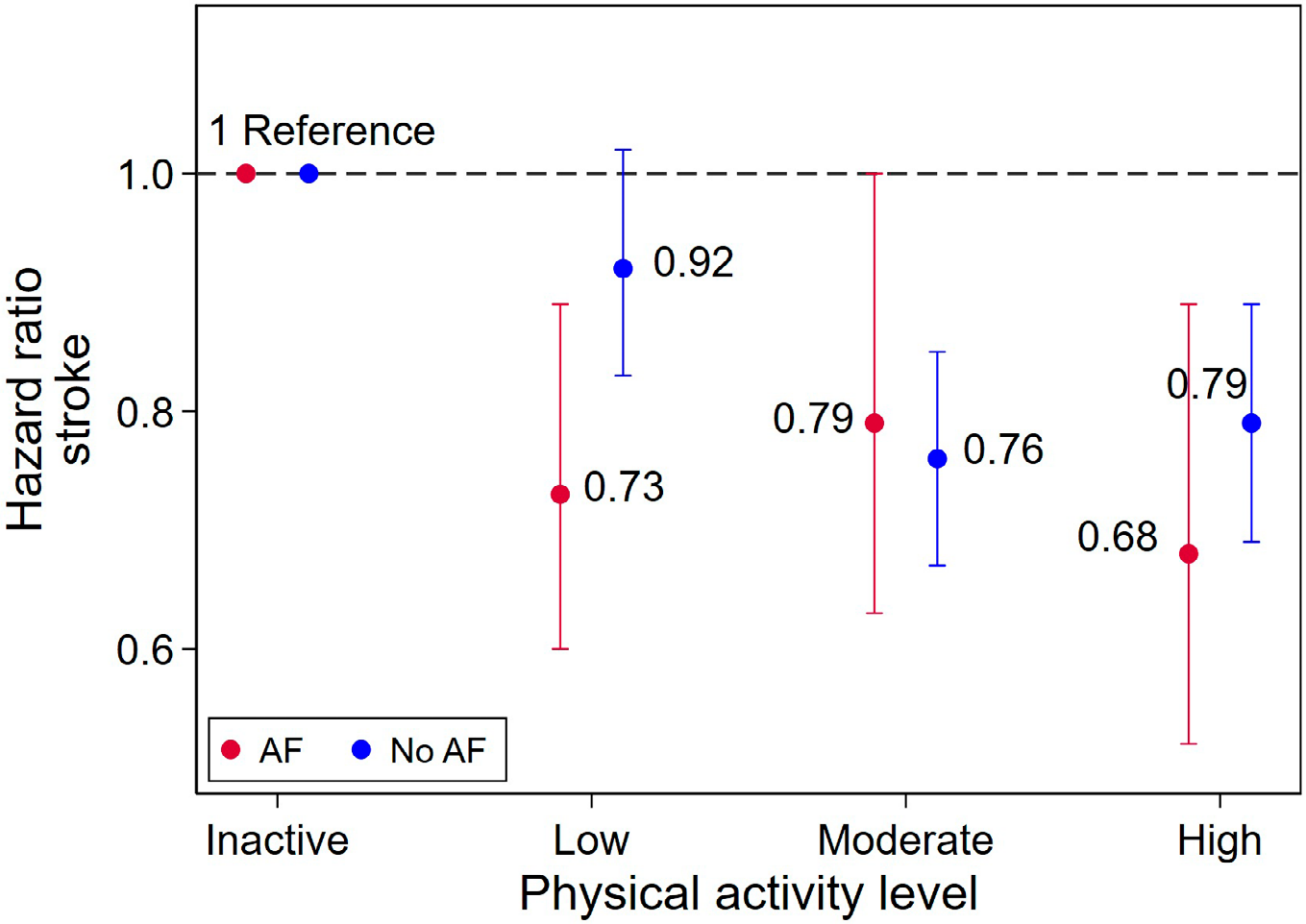
HR and corresponding 95% CI for the outcome stroke according to PA level in individuals with and without AF. HRs were estimated with a flexible parametric (proportional hazards) model that parameterized the baseline hazard function with restricted cubic splines with knots placed at the 25th, 50th and 75th centile of the uncensored log event time. The model was adjusted for age, sex, body mass index, education, smoking status, frequency of alcohol consumption, coronary heart disease, and study cohort. AF indicates atrial fibrillation; HR, hazard ratio; and PA, physical activity.

We observed similar associations when analyzing ischemic stroke only (Table S10). PA was not significantly associated with intracerebral hemorrhage, although a 37% (95% CI, −20% to 67%) risk reduction was indicated in highly active individuals with AF.

### PA and All‐Cause Death

In total, 5 600 (6.9%) participants without and 2 233 (34.2%) participants with AF died during follow‐up. The absolute risk of death after 15 years was 9.9% (95% CI, 9.6%–10.2%) in individuals without and 48.5% (95% CI, 47.2%–49.9%) in individuals with AF, respectively. An 11% to 22% relative mortality risk reduction was observed for low to high PA in the total cohort, with similar risk reductions observed in individuals with and without AF (*P* interaction=0.484). Furthermore, in individuals with AF (Figure 4), low, moderate, and high PA increased the life expectancy by 0.50 (95% CI, 0.22–0.78), 0.66 (95% CI, 0.31–1.01) and 1.15 (95% CI, 0.77–1.53) years, respectively, compared with being inactive. The standardized and time‐dependent HRs according to PA level (Figure 5) indicated a similar pattern in individuals with and without AF, with an attenuated effect of PA toward the end of the follow‐up period.

**Figure 4:**
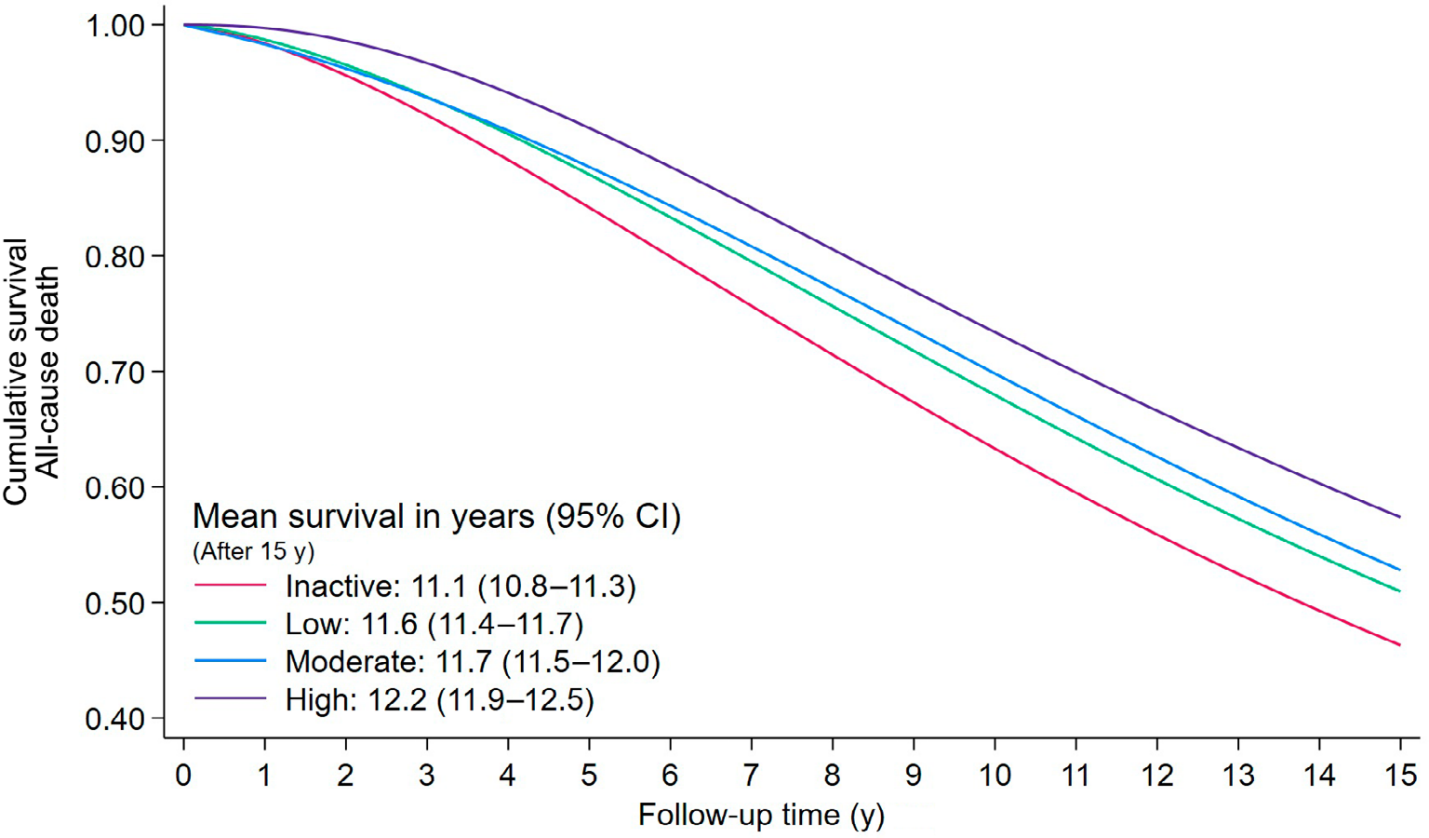
Standardized survival function for the outcome all‐cause death in individuals with atrial fibrillation and according to PA level. Mean survival (RMST) is given after 15 years of follow‐up. The survival function was constructed after fitting a flexible parametric survival model (adjusted for age, sex, body mass index, education, smoking, frequency of alcohol consumption, coronary heart disease, and study cohort) that parameterized the baseline hazard function with restricted cubic splines with knots placed at the 25th, 50th, and 75th centile of the uncensored log event time. Furthermore, PA was modeled as a time‐dependent covariate with 2 *df*; that is, the time‐dependent effect of PA was modeled with restricted cubic splines with a knot placed at the 50th centile of the uncensored log event time. Regression standardization was subsequently applied to construct the survival curves and to estimate the RMST. AF indicates atrial fibrillation; PA, physical activity; and RMST, restricted mean survival time.

**Figure 5:**
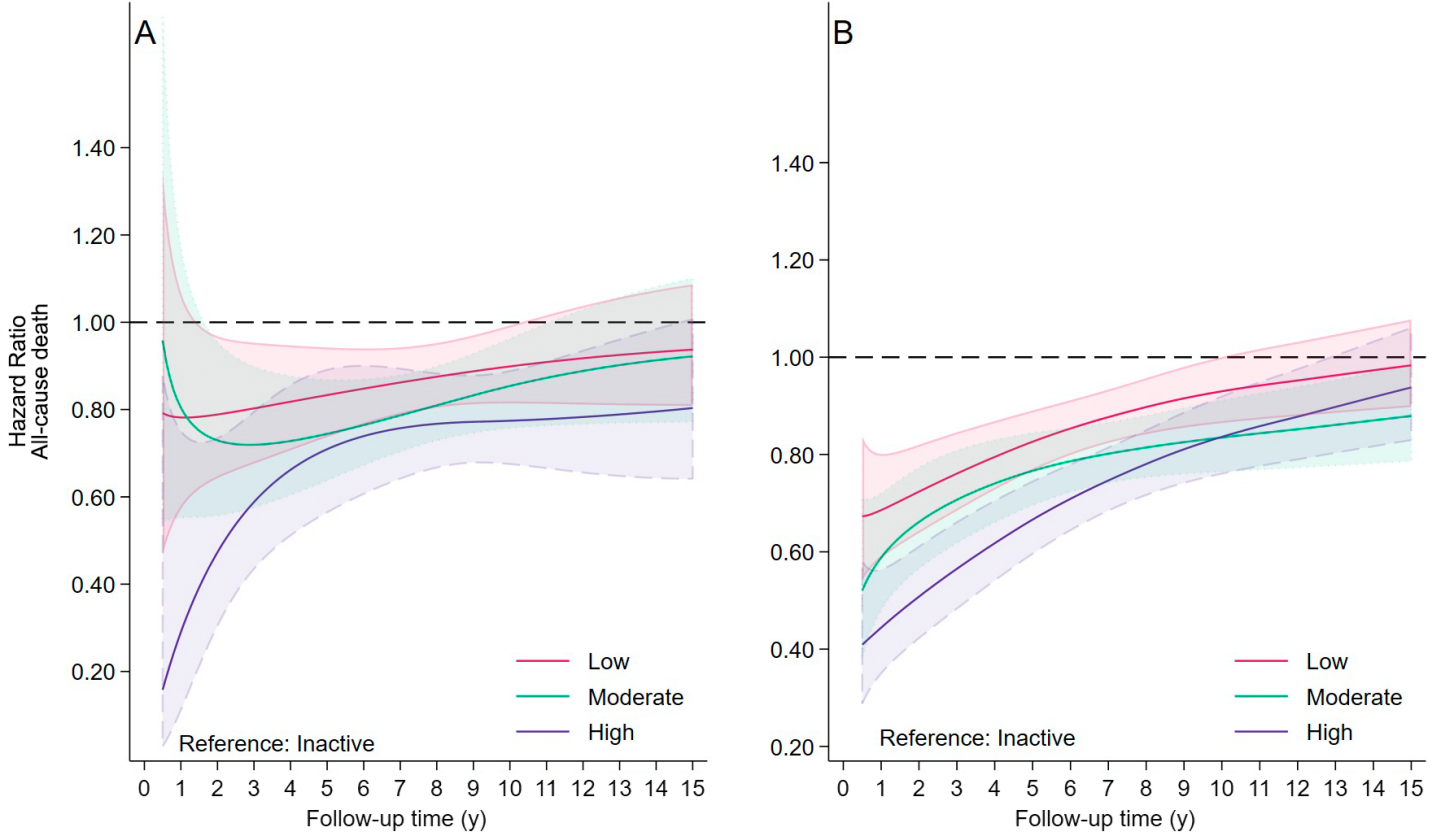
Time‐dependent HR and corresponding 95% CI for all‐cause mortality according to PA level in (A) individuals with AF and (B) individuals without AF. The time‐dependent HRs for death were estimated in separate groups by AF status with a flexible parametric model that was adjusted for age, sex, body mass index, education, smoking, frequency of alcohol consumption, coronary heart disease, and study cohort. The baseline hazard function was parameterized with restricted cubic splines with knots placed at the 25th, 50th, and 75th centile of the uncensored log event time. Furthermore, an interaction between PA and time was modeled with 2 *df*; that is, the time‐dependent effect of PA was modeled with restricted cubic splines with a knot placed at the 50th centile of the uncensored log event time. For illustrative purposes, the HR before 0.5 years is not displayed. AF indicates atrial fibrillation; HR, hazard ratio; and PA, physical activity.

### Sensitivity Analyses

Additional adjustment for the CHA_2_DS_2_‐VA score (Table S11) or use of oral anticoagulants (Table S12) did not markedly change our estimates. Furthermore, in the subanalysis of individuals at low, intermediate, and high risk of stroke according to the CHA_2_DS_2_‐VA score, point estimates suggested a beneficial effect of PA across all subgroups, however, the CIs were wide in the low and intermediate risk groups (Table S13). Reanalyzing the data after conducting multivariate imputation (Table S14) and after excluding prevalent AF cases or after censoring incident AF cases (Table S15) corroborated the main findings. In the total cohort, exclusion of individuals self‐reporting coronary heart disease (Table S16) and exclusion of the two first  years of follow‐up (Table S17) did not impact the results substantially. Self‐reported PA did not correlate well with light activity (AF: Spearman's ρ=0.02, no AF: Spearman's ρ=0.05) but was modestly correlated with moderate to vigorous PA as assessed by accelerometery (AF: Spearman's ρ=0.34, no AF: Spearman's ρ=0.32) (Table S18). Furthermore, a general increase in most accelerometer‐derived parameters was observed with increasing levels of self‐reported PA in individuals with and without AF (Table S19).

The association between PA and stroke was not modified by age or sex in the total cohort or among individuals with AF. However, for the outcome all‐cause mortality, sex‐specific analyses (Table S20) indicated that the risk reduction associated with PA was larger among women (*P* interaction=0.074). Furthermore, in individuals with AF, effect modification by age (*P* interaction=0.024) was observed, suggesting a larger risk reduction among younger individuals (Table S21).

## Discussion

After 15 years of follow‐up, PA was associated with a reduced risk of stroke and mortality, with similar benefits observed irrespective of AF status. Positive associations were documented even among individuals performing low levels of PA, although the largest benefit was observed at moderate to high levels of PA. Furthermore, in individuals with AF, high PA was associated with an increased life expectancy of >1 year.

### PA and Stroke

We observed a beneficial effect of PA on the risk of stroke, which broadly aligns with previously published meta‐analyses.^42^, ^43^ Furthermore, our results suggest that any level of PA beyond inactivity is beneficial for individuals with AF.

Given that individuals with AF have an increased stroke risk, our findings provide important data on the relationship between PA and stroke in individuals with AF. Previous observations have shown a beneficial effect of PA on cardiovascular mortality and heart failure, but with somewhat inconclusive results regarding the association with stroke.^17^, ^18^, ^19^, ^20^ Conversely, our results suggest that individuals with AF may expect similar benefits of PA as individuals without AF and further indicate that even low PA levels have a preventive effect against stroke. Thus, while the recently published European Society of Cardiology AF guidelines highlight exercise as part of the comorbidity and risk factor management in individuals with AF,^10^ the results of our study provide an argument to add PA as a complementary option to conventional AF treatment strategies to prevent stroke.

We observed that the estimated risk reduction for stroke was less pronounced compared with the relative rate, particularly for highly active individuals with AF. This is likely attributed, at least in part, to the large mortality rate observed in inactive individuals with AF. It is well documented that in the presence of competing risks, the 1:1 relationship between the risk and the rate is lost because the rate of the competing event also impacts the risk of the outcome of interest.^38^, ^40^ This may cause a discrepancy between the cause‐specific hazard rate and the cause‐specific cumulative incidence, particularly when the rate of the competing event is high, and is associated with the exposure of interest.^38^ Thus, the beneficial impact of PA on the rate of stroke further strengthens the argument of PA as a preventive measure in individuals with AF.

### PA and Mortality

In line with previous studies, we observed that the risk of mortality was lower in physically active individuals, a finding that was consistent regardless of AF status. Although randomized studies with short‐term exercise interventions indicate that high‐intensity PA is both safe and beneficial for patients with AF,^44^ the optimal dose of PA remains unclear. Previous studies have shown that compared with inactive individuals, low to high PA levels are associated with an 18% to 55% mortality rate reduction in individuals with AF.^18^, ^19^, ^20^ Our study corroborates previous literature and suggests that even low levels of PA are beneficial. Furthermore, these results adds valuable knowledge by demonstrating that the highest level of PA is associated with a more pronounced mortality rate reduction compared with low and moderate PA, and is in line with a recent study from the UK Biobank.^20^ Moreover, our study adds to previous literature by quantifying the difference in life expectancy associated with PA. We observed that low, moderate, and high levels of PA were associated with a 0.5‐, 0.7‐, and 1.2‐year increase in life expectancy, indicating improved longevity in active individuals with AF. Importantly, the mean survival time is dictated by the length of the follow‐up period,^41^ and it is therefore likely that the number of life years gained may be increased with a longer exposure period (provided that the level of PA is maintained). This is consistent with Ahn and colleagues,^19^ who reported that engaging in PA before an AF diagnosis was associated with a greater mortality rate reduction (HR, 0.61) than initiating PA following an AF diagnosis (HR, 0.82). Collectively, this may provide an argument for 2 distinct messages that could be disseminated to individuals with AF and other relevant stakeholders: (1) The earlier exercise is initiated, the larger the benefit; and (2) regardless of when PA is initiated, substantial health benefits are to be expected.

### Strengths and Limitations

Our study has several strengths. We conducted a prospective cohort study from the 2 largest population‐based studies in Norway, which have a high participation proportion and generalizability to a general Western population. Furthermore, these cohorts provide large amounts of information, which allowed us to adjust for multiple confounding factors and conduct several sensitivity analyses to determine the robustness of our findings. The main strength of the present investigation is the application of gold‐standard methods to classify AF^26^, ^27^ as well as linkage with national registries for ascertainment of incident stroke, mortality, and AF. Another strength is the long follow‐up period, securing a high number of outcomes and enabling us to draw more firm conclusions. Another strength is that individuals with incident AF during follow‐up was reclassified, which reduces misclassification and may improve our ability to make statistical inferences among individuals with AF.

Our study has limitations that should be addressed. The main limitation is that we are unable to rule out the possibility of unmeasured or residual confounding due to our observational study design. Thus, data from large‐scale randomized controlled trials are warranted to further guide exercise recommendations for the prevention of stroke in individuals with AF. Another limitation is that PA was self‐reported. The questionnaire used in the present study is moderately correlated with device‐measured moderate to vigorous PA and maximal oxygen consumption but correlates poorly with low‐intensity PA and total energy expenditure.^31^, ^32^ However, it has previously been shown that self‐reported PA generally agrees with accelerometery and is able to rank individuals in coarse PA categories.^32^ We observed the same in this study, regardless of AF status. Moreover, a lack of information on anticoagulation for the entire study cohort limits our ability to account for this potential confounder in the main analyses. However, in our subgroup analysis of participants from the Tromsø Study, adjustment for anticoagulants did not impact the estimated effect of PA. Although we could not ascertain updated information on anticoagulant use in individuals diagnosed with AF after the baseline examination. Another limitation is that this study was likely underpowered to observe statistically significant and clinically meaningful associations between PA and stroke subtypes among individuals with AF. Furthermore, we did not estimate the impact of dimensions of PA such as frequency, intensity, and duration. Because these dimensions are closely related, bias due to confounding or conditioning on a mediator or a collider is a concern. Therefore, untangling the impact of each dimension of PA is most likely more appropriate with a randomized controlled design or a longitudinal observational design with a sophisticated analytical methodology.

## Conclusions

In conclusion, our study suggests that PA is associated with similar effects on stroke and all‐cause mortality in individuals with and without AF. Even the transition from inactivity to low levels of PA appears beneficial, although the largest stroke and mortality risk reductions are observed in the most active individuals. Our study provides data supporting that PA may complement current treatment strategies to prevent stroke in patients with AF and underlines that PA is associated with increased longevity in individuals with AF.

## Sources of Funding

K.R.J. was funded by the Northern Norway Regional Health Authority (Grant No. HNF1568‐21).

## Disclosures

M.M. has received lecture fees from AstraZeneca, Bayer, Boehringer‐Ingelheim, Bristol Myers Squibb, MSD, and Pfizer. Data from the Norwegian Patient Register has been used in this publication. The interpretation and reporting of these data are the sole responsibility of the authors, and no endorsement by the Norwegian Patient Registry is intended nor should be inferred.

## Supporting information

Figures S1–S3
Tables S1–S21
Supplemental Analyses
References 45–50

STROBE Statement
