# Supplementary material for: Impact of Physical Activity on Stroke and Mortality in Individuals With and Without Atrial Fibrillation: The HUNT Study and the Tromsø Study
Source: J Am Heart Assoc. 2026 Aug 5;15(16):e048812. doi: 10.1161/JAHA.126.048812 (PMC13695750; doi:10.1161/JAHA.126.048812)
Supplement: Supplementary file 2 [file JAH3-15-e048812-s002.pdf]

Supplemental Material

Supplemental Analyses

Consideration of the proportional hazards assumption for the outcome stroke

The -ln(-ln) plot (figure S2) may indicate that there is a degree of non-proportionality initially, however the overall plot appears reasonable in terms of the PH assumption. Schoenfeld's test (table S6) shows that there is no statistically significant departure from the PH assumption for any level of PA. This is corroborated by a test of interaction between levels of PA and follow-up time. Thus, based on these results, we concluded that the PH assumption was met for the outcome stroke.

Consideration of the proportional hazards assumption for the outcome all-cause mortality

The -ln(-ln) plot (figure S3) does not display crossing of the curves. However, the curves appear to be converging toward the end of the follow-up period. Furthermore, the test of Schoenfeld's residuals (table S7) indicates that the PH assumption is violated for all levels of PA. Further evaluation of an interaction with time corroborates this notion. Thus, we concluded that the PH assumption was not met for the outcome all-cause mortality.

Table S1.

Overview of organization of the 3 questions used to categorize participants into physical activity groups.

Frequency	Intensity	Duration
**How frequently do you exercise?**	**If you do such exercise as frequently as once or more times a week: How hard do you push yourself?**	**How long does each session last**
Never (0)	I take it easy without breaking a sweat (moderate) (3)	Less than 15 minutes (7.5)
Less than once a week (0.5)	I push myself so hard that I lose my breath and break into a sweat (vigorous) (6)	15-29 minutes (22)
Once a week (1)	I push myself to near exhaustion (very vigorous) (9)	30 minutes to 1 hour (45)
2-3 times a week (2.5)		More than 1 hour (60)
Almost every day (5)		

Categorisation of physical activity level with example calculation:

**Inactive (0)**	**Low (1-449)**		**Moderate (450-899)**		**High (≥900)**	
	Vigorous exercise less than once a week for 15-29 minutes	0.5•6•22 = 66	Moderate exercise 2-3 times a week, more than 1 hour	2.5•3•60 = 450	Moderate exercise almost every day for more than 1 hour	5•3•60 = 900
	Moderate exercise almost every day for less than 15 minutes	5•3•7.5 = 112.5	Moderate exercise almost every day for 30 minutes to 1 hour	5•3•45 = 675	Vigorous exercise almost every day for 30 minutes to 1 hour	5•6•45 = 1350
	Moderate exercise 2-3 times a week for 30 minutes to 1 hour	2.5•3•45 = 337.5	Vigorous exercise 2-3 times a week for 30 minutes to 1 hour	2.5•6•45 = 675	Very vigorous exercise almost every day for more than 1 hour	5•9•60 = 2700

The numbers in brackets indicate the scores given for each alternative. The product of the scores from the 3 questions were taken to calculate the total metabolic equivalent of task (MET)-score as previously described[32]. The scores assigned for the frequency category was taken to determine the average weekly frequency of physical activity, while the MET scores were based on previous literature where 3-5.9 METs is considered moderate activity, 6 METs is considered vigorous activity and 9 METs may be considered as very vigorous activity[45, 46]. The scores for duration were taken as the median of each alternative given, except for 'more than 1 hour' where a conservative estimate of 60 minutes where set. Individuals who replied that they never exercised or replied that they exercised but did not report duration of exercise were assigned to the inactive category.

Table S2.
Overview of organization of the covariate 'Smoking'.

	Never smoker	Previous smoker	Current smoker
**Tromsø6**	Never smoker	Previous smoker	Current smoker
**Tromsø7**	Never smoker	Previous smoker	Current smoker
**HUNT3**	Never smoker	Previous smoker<br>Previously smoked sometimes	Current smoker<br>Smoke sometimes
**HUNT4**	Never smoker	Previous smoker<br>Previously smoked sometimes	Current smoker<br>Smoke sometimes

In the HUNT study there were 2 additional categories compared with the Tromsø Study. Therefore, the categories were merged into three distinct categories: [1] Never smoker, [2] previous smoker, [3] current smoker.

Table S3.
Overview of organization of the covariate 'frequency of alcohol consumption'.

	Not in the last year	Monthly or less	2-4 times per month	2-3 times per week	4 or more times per week
**Tromsø6**	Never	Monthly or less	2-4 times per month	2-3 times per week	4 times or more times per week
**Tromsø7**	Never	Monthly or less	2-4 times per month	2-3 times per week	4 times or more per week
**HUNT3**	I have never had any alcohol<br>Not in the last year	A few times per year<br>1 time per month	2-3 times per month<br>Once per week	2-3 times per week	4-7 times per week
**HUNT4**	I have not had any alcohol in the last 12 months<br>I have never had any alcohol	1 time per month or less	2-4 times per month	2-3 times per week	4 or more times per week

There were more categories in the HUNT Study compared to the Tromsø Study. Therefore, we harmonized the alcohol question into 5 categories based on the statements above.

Table S4.
Overview of organization of the covariate 'University education'.

	Yes	No
**Tromsø6**	College/University <4 years<br>College/University ≥4 years	Primary school/Secondary school<br>Vocational upper secondary school/Vocational school<br>General upper secondary school or gymnasium
**Tromsø7**	College/University <4 years<br>College/University ≥4 years	Primary school/Secondary school<br>Vocational upper secondary school/Vocational school<br>General upper secondary school or gymnasium
**HUNT3**	Administrative leader or politician<br>Academic profession<br>Profession requiring max 3 years of college	Military/unspecified<br>Office or customer relation<br>Sales, service and care professions<br>Agriculture, forestry, or fisheries profession<br>Artisan or similar<br>Process- and machine operator, transport worker or similar<br>Profession without education requirements
**HUNT4**	College/University <4 years<br>College/University ≥4 years	Primary school/Secondary school<br>1-2 years of upper secondary school<br>3 years of upper secondary school<br>Vocational qualification certificate/Trainee letter

Because there was no education question available in the HUNT3 study, we used the question regarding occupation to obtain a proxy for socioeconomic status and made the assumption that participants who reported an occupation as 'administrative leader or politician', 'academic profession' and 'profession requiring max 3 years of college' would be likely to have higher education whereas the other categories would be less likely to have higher education.

Table S5.

Overview of how the CHA_2DS_2-VA score was calculated.

	**Risk score:**
**Age**	<65: 0<br>65-74: 1<br>≥75: 2
**Heart failure**<br>Self-reported	1
**Hypertension**<br>Self-reported current/previous use of blood pressure medication and/or systolic blood pressure >139 and/or diastolic blood pressure of >89 mmHg	1
**History of stroke**<br>Self-reported	2
**Coronary heart disease**<br>Self-reported Angina or heart attack	1
**Diabetes**<br>Self-reported	1
**Calculation of the CHA_2DS_2-VA risk score**	0 points = Low risk<br>1 point = Intermediate risk<br>≥2 points = High risk

Description of the calculation of the CHA_2DS_2-VA score. We calculated the score based on the covariates described above and categorized participants into three categories 'Low risk', 'Intermediate risk' and 'High risk'. Notably, when we adjusted for the risk score in sensitivity analyses, it was modelled as a continuous covariate on a scale from 0-8. Information regarding heart failure was not available in Tromsø6.

Table S6.

Test of Schoenfeld's residuals and tests of interaction with time by level of physical activity for the outcome stroke.

	**Test of Schoenfeld's residuals**	**Test for interaction with follow-up time**
Physical activity level	*P-value*	*P-value*
Inactive	.	.
Low	0.99	0.98
Moderate	0.95	0.83
High	0.74	0.97

Table S7.

Test of Schoenfeld's residuals and tests of interaction with time by level of physical activity for the outcome all-cause mortality.

	**Test of Schoenfeld's residuals**	**Test for interaction with follow-up time**
Physical activity level	*P-value*	*P-value*
Inactive	.	.
Low	0.007	0.009
Moderate	0.010	0.038
High	<0.001	<0.001

Table S8.

Descriptive characteristics according to physical activity level in participants with atrial fibrillation.

	Inactive (n = 1 358)	Low (n = 2 676)	Moderate (n = 1 378)	High (n = 1 127)
Age	68.8 (11.9)	67.7 (10.9)	66.4 (10.6)	64.5 (11.1)
Female	506 (37.3)	1 075 (40.2)	495 (35.9)	305 (27.1)
Male	852 (62.7)	1 601 (59.8)	883 (64.1)	822 (72.9)
MET/min per week	0.0	210.1 (114.4)	652.4 (66.2)	1 233.6 (385.4)
Height (cm)	171.0 (9.9)	171.2 (9.5)	172.6 (9.4)	174.2 (9.0)
Weight (kg)	86.6 (16.9)	83.2 (15.6)	82.7 (15.0)	82.6 (14.1)
BMI (kg/m^2)	29.6 (5.0)	28.3 (4.5)	27.7 (4.2)	27.1 (3.7)
University educated	266 (19.6)	796 (29.7)	533 (38.7)	452 (40.1)
Smoking status				
Never	414 (30.5)	960 (35.9)	492 (35.7)	462 (41.0)
Previously	596 (43.9)	1 231 (46.0)	687 (49.9)	551 (48.9)
Currently	348 (25.6)	485 (18.1)	199 (14.4)	114 (10.1)
Frequency of alcohol consumption				
Not in the last year	267 (19.7)	376 (14.1)	141 (10.2)	97 (8.6)
Monthly or less	464 (34.2)	856 (32.0)	377 (27.4)	298 (26.4)
2-4 times per month	442 (32.5)	982 (36.7)	503 (36.5)	424 (37.6)
2-3 times per week	135 (9.9)	354 (13.2)	287 (20.8)	239 (21.2)
4 or more times per week	50 (3.7)	108 (4.0)	70 (5.1)	69 (6.1)
Systolic blood pressure (mmHg)	139.4 (20.9)	139.6 (21.5)	139.1 (21.3)	137.8 (20.5)
Diastolic blood pressure (mmHg)	76.1 (12.2)	76.5 (11.7)	77.1 (11.6)	77.0 (10.9)
Resting heart rate (beats/min)	70.0 (14.1)	68.2 (12.9)	67.1 (12.2)	64.5 (12.2)
Total cholesterol (mmol/L)	5.4 (1.2)	5.4 (1.1)	5.5 (1.1)	5.4 (1.1)
High density lipoprotein (mmol/L)	1.3 (0.3)	1.3 (0.4)	1.4 (0.4)	1.4 (0.4)
Triglycerides (mmol/L)	1.9 (1.1)	1.7 (1.0)	1.6 (0.9)	1.5 (0.8)
Blood pressure medication	756 (56.8)	1 276 (48.4)	591 (44.1)	412 (37.4)
Anticoagulants[a]	37 (18.6)	128 (15.5)	50 (13.4)	40 (12.6)
Antiplatelets[a]	67 (33.7)	183 (22.1)	84 (22.5)	49 (15.5)
Coronary heart disease	299 (22.0)	499 (18.6)	222 (16.1)	138 (12.2)
Heart failure[b]	104 (8.9)	130 (6.5)	47 (4.4)	46 (5.1)
Diabetes	175 (13.0)	254 (9.5)	103 (7.5)	62 (5.5)
CHA_2DS_2-VA score[b]				
Low risk	150 (13.0)	260 (13.0)	176 (16.6)	173 (19.6)
Intermediate risk	233 (20.2)	443 (22.2)	266 (25.1)	227 (25.7)
High risk	771 (66.8)	1 291 (64.7)	619 (58.3)	484 (54.8)

Descriptive characteristics are given for participants who were prevalent with AF at baseline or developed AF during follow-up. Participants were classified according to weekly PA level. Inactive: 0 MET minutes per week; Low: 1-449 MET minutes per week; Moderate: 450-899 MET minutes per week; High: ≥900 MET minutes per week. [a]Information only available in the Tromsø Study. [b]Information on heart failure not available in the Tromsø6 study cohort. Values are presented as mean ± SD for continuous variables and as numeric (%) for categorical variables. AF: Atrial fibrillation, PA: Physical activity, MET: Metabolic equivalent of task, BMI; Body mass index.

Table S9.

Descriptive characteristics according to physical activity level in participants without atrial fibrillation.

	Inactive (n = 13 946)	Low (n = 30 079)	Moderate (n = 19 144)	High (n = 17 632)
Age	49.0 (15.6)	51.1 (14.4)	49.8 (13.9)	47.5 (14.6)
Female	5 978 (42.9)	16 668 (55.4)	11 674 (61.0)	9 243 (52.4)
Male	7 968 (57.1)	13 411 (44.6)	7 470 (39.0)	8 389 (47.6)
MET/min per week	0.0 (0.0)	222.8 (109.2)	659.4 (54.7)	1 288.9 (412.6)
Height (cm)	172.2 (9.5)	170.6 (9.3)	170.7 (9.0)	172.1 (9.0)
Weight (kg)	82.9 (17.4)	79.9 (16.0)	78.0 (15.0)	77.6 (14.6)
BMI (kg/m^2)	27.9 (5.1)	27.4 (4.6)	26.7 (4.3)	26.1 (4.0)
University educated	3 663 (26.3)	10 789 (35.9)	8 694 (45.4)	7 951 (45.1)
Smoking status				
Never	4 863 (34.9)	12 459 (41.4)	8 904 (46.5)	8 718 (49.4)
Previously	4 552 (32.6)	10 926 (36.3)	7 314 (38.2)	6 646 (37.7)
Currently	4 531 (32.5)	6 694 (22.3)	2 926 (15.3)	2 268 (12.9)
Frequency of alcohol consumption				
Not in the last year	1 400 (10.0)	2 718 (9.0)	1 267 (6.6)	1 055 (6.0)
Monthly or less	4 965 (35.6)	9 289 (30.9)	5 208 (27.2)	4 939 (28.0)
2-4 times per month	5 678 (40.7)	12 858 (42.7)	8 604 (44.9)	7 818 (44.3)
2-3 times per week	1 540 (11.0)	4 307 (14.3)	3 432 (17.9)	3 178 (18.0)
4 or more times per week	363 (2.6)	907 (3.0)	633 (3.3)	642 (3.6)
Systolic blood pressure (mmHg)	129.1 (18.1)	128.9 (18.9)	126.8 (18.3)	126.4 (17.8)
Diastolic blood pressure (mmHg)	74.1 (11.0)	74.3 (10.9)	73.3 (10.6)	72.9 (10.7)
Resting heart rate (beats/min)	73.0 (11.8)	70.8 (11.6)	68.7 (11.4)	66.9 (11.3)
Total cholesterol (mmol/L)	5.4 (1.1)	5.5 (1.1)	5.4 (1.1)	5.3 (1.1)
High density lipoprotein (mmol/L)	1.3 (0.3)	1.4 (0.4)	1.4 (0.4)	1.5 (0.4)
Triglycerides (mmol/L)	1.8 (1.2)	1.6 (1.0)	1.5 (0.9)	1.4 (0.9)
Blood pressure medication	2 406 (20.7)	5 543 (21.0)	2 920 (17.7)	2 214 (15.1)
Anticoagulants[a]	.	.	.	.
Antiplatelets[a]	138 (11.4)	689 (7.4)	318 (5.9)	238 (4.6)
Coronary heart disease	698 (5.0)	1 314 (4.4)	641 (3.3)	539 (3.1)
Heart failure[b]	97 (0.7)	135 (0.5)	79 (0.5)	51 (0.3)
Diabetes	613 (4.4)	1 254 (4.2)	620 (3.2)	458 (2.6)
CHA_2DS_2-VA score[b]				
Low risk	5 925 (53.6)	11 761 (53.6)	8 432 (58.9)	7 869 (61.3)
Intermediate risk	2 985 (27.0)	5 834 (26.6)	3 617 (25.3)	3 180 (24.8)
High risk	2 135 (19.3)	4 328 (19.7)	2 275 (15.9)	1 784 (13.9)

Descriptive characteristics according to PA level. Inactive: 0 MET minutes per week; Low: 1-449 MET minutes per week; Moderate: 450-899 MET minutes per week; High: ≥900 MET minutes per week. [a]Information only available in the Tromsø Study. [b]Information on heart failure not available in the Tromsø6 Study cohort. Values are presented as mean ± SD for continuous variables and numeric (%) for categorical variables. PA: Physical activity, MET: Metabolic equivalent of task, BMI: Body mass index.

Table S10.

Risk of ischemic stroke and intracerebral haemorrhage.

	AR (95% CI)	RR (95% CI)
**Total sample**		
**Ischemic stroke**		
Inactive	0.053 (0.049, 0.057)	1 Reference
Low	0.047 (0.045, 0.050)	0.90 (0.82, 0.99)
Moderate	0.042 (0.039, 0.046)	0.81 (0.72, 0.90)
High	0.042 (0.038, 0.046)	0.79 (0.71, 0.89)
**Intracerebral haemorrhage**		
Inactive	0.007 (0.006, 0.009)	1 Reference
Low	0.007 (0.006, 0.008)	1.00 (0.78, 1.29)
Moderate	0.006 (0.005, 0.007)	0.82 (0.60, 1.12)
High	0.007 (0.006, 0.009)	1.06 (0.77, 1.44)
**Individuals with AF**		
**Ischemic stroke**		
Inactive	0.161 (0.137, 0.189)	1 Reference
Low	0.129 (0.114, 0.146)	0.80 (0.66, 0.98)
Moderate	0.147 (0.125, 0.174)	0.92 (0.73, 1.15)
High	0.136 (0.111, 0.167)	0.84 (0.65, 1.10)
**Intracerebral haemorrhage**		
Inactive	0.035 (0.024, 0.050)	1 Reference
Low	0.028 (0.021, 0.038)	0.80 (0.51, 1.26)
Moderate	0.024 (0.016, 0.038)	0.70 (0.39, 1.23)
High	0.022 (0.013, 0.037)	0.63 (0.33, 1.20)

All models were adjusted for age, sex, body mass index, education, smoking status, frequency of alcohol consumption, coronary heart disease and study cohort. AF: Atrial fibrillation, AR: Absolute risk, RR: Risk ratio, CI: Confidence interval.

Table S11.

Risk ratio and confidence interval for stroke by PA level after additional adjustment for the CHA_2DS_2-VA score in individuals with AF.

	RR (95% CI) [a]	RR (95% CI) [b]
Inactive	1 Reference	1 Reference
Low	0.85 (0.70, 1.04)	0.80 (0.67, 0.95)
Moderate	0.84 (0.66, 1.08)	0.87 (0.71, 1.07)
High	0.90 (0.69, 1.17)	0.80 (0.63, 1.01)

[a]Adjusted for age, sex, body mass index, education, smoking status, frequency of alcohol consumption, study cohort and CHA_2DS_2-VA score.

[b]Adjusted for age, sex, body mass index, education, smoking status, frequency of alcohol consumption, coronary heart disease, study cohort, diabetes, self-reported stroke and blood pressure medication. Heart failure was not available in Tromsø6. Therefore, we modelled the effect of stroke with adjustment for the CHA_2DS_2-VA score after excluding these participants, and subsequently also included them in a second analysis where we adjusted for all components of the score except for heart failure.

Table S12.

Risk ratio and confidence interval for stroke in individuals with AF with and without additional adjustment for oral anticoagulants in AF participants in the Tromsø Study.

	RR (95% CI)[a]	RR (95% CI)[b]
Inactive	1 reference	1 Reference
Low	0.64 (0.45, 0.90)	0.63 (0.45, 0.90)
Moderate	0.87 (0.60, 1.28)	0.87 (0.59, 1.27)
High	0.48 (0.28, 0.82)	0.47 (0.28, 0.81)

[a]Adjusted for age, sex, body mass index, education, smoking status, frequency of alcohol consumption, coronary heart disease and study cohort, [b]additionally adjusted for anticoagulants. Oral anticoagulants adjusted for: Warfarin, Apixaban, Dabigatran etexilate and Rivaroaxaban. Only participants who attended Tromsø6 and Tromsø7 was included in this analysis because information regarding anticoagulants was not available for participants that attended HUNT3 or HUNT4. Furthermore, we were unable to ascertain updated information regarding use of anticoagulants for individuals diagnosed with AF during follow-up (n = 1 209). RR: Risk ratio, CI: Confidence interval, AF: Atrial fibrillation, PA: Physical activity.

Table S13.

Crude stroke and mortality rates in individuals with AF and corresponding absolute and relative risks of the composite outcome stroke or mortality in individuals categorised as at either low, intermediate or high risk of stroke according to their baseline CHA_2DS_2-VA score.

	Crude stroke rate per 100 000 (95% CI)	Crude mortality rate per 100 000 (95% CI)	AR Stroke/Mortality (95% CI)	RR Stroke/Mortality (95% CI)
**Low risk**				
Inactive	986 (493, 1971)	1 772 (1 068, 2 939)	0.250 (0.173, 0.360)	1 Reference
Low	978 (579, 1651)	737 (408, 1 330)	0.193 (0.136, 0.276)	0.77 (0.47, 1.26)
Moderate	384 (144, 1024)	764 (382, 1 528)	0.139 (0.082, 0.235)	0.56 (0.30, 1.04)
High	512 (213, 1230)	809 (405, 1 618)	0.158 (0.097, 0.260)	0.63 (0.34, 1.17)
**Intermediate risk**				
Inactive	902 (523, 1 553)	2 830 (2091, 3829)	0.366 (0.297, 0.451)	1 Reference
Low	748 (483, 1 160)	2 531 (2002, 3199)	0.341 (0.288, 0.402)	0.93 (0.72, 1.20)
Moderate	1108 (679, 1808)	1 844 (1273, 2671)	0.315 (0.249, 0.399)	0.86 (0.63, 1.17)
High	771 (415, 1 433)	1 404 (895, 2200)	0.247 (0.181, 0.337)	0.67 (0.46, 0.98)
**High risk**				
Inactive	2 836 (2 364, 3 401)	10 136 (9 240, 11 118)	0.674 (0.639, 0.711)	1 Reference
Low	2 313 (1 982, 2 699)	8 711 (8 068, 9 406)	0.623 (0.593, 0.654)	0.92 (0.87, 0.98)
Moderate	2 174 (1 734, 2 726)	7 662 (6 818, 8 610)	0.606 (0.567, 0.648)	0.90 (0.83, 0.97)
High	2 102 (1 639, 2 696)	5 750 (4 968, 6 654)	0.552 (0.507, 0.600)	0.82 (0.75, 0.90)

All models were adjusted for age, sex, body mass index, education, smoking status, frequency of alcohol consumption and study cohort. AR: Absolute risk, RR: Risk ratio, CI: Confidence interval. A proportional hazards model was fitted to the data, and coronary heart disease was not adjusted for to achieve model convergence.

Table S14.

Hazard ratio and confidence interval for stroke in participants with AF after conducting multivariate imputation with chained equations.

	**Stroke** HR (95% CI)
Inactive	1 Reference
Low	0.80 (0.67, 0.97)
Moderate	0.85 (0.69, 1.06)
High	0.72 (0.55, 0.92)

HR was adjusted for age, sex, body mass index, education, smoking status, frequency of alcohol consumption, coronary heart disease and study cohort. The model was estimated after using a multivariate chained equations approach to impute missing values of covariates in the model. 25 Datasets were imputed. Imputation was conducted with Statas “mi impute” function. For the imputation model we included all covariates of interest with missing data and the outcome (the event indicator and the Nelson-Aalen cumulative hazard function) as recommended[47]. Furthermore, we included variables that predicted one or more of the covariates as ancillary variables to increase the plausibility of the missing at random assumption[48]. AF: Atrial fibrillation, HR: Hazard ratio, CI: Confidence interval.

Table S15.

Risk ratio for stroke and mortality in individuals with incident and prevalent AF.

**Individuals with prevalent AF**	**Stroke** RR (95% CI)	**All-cause mortality** RR (95% CI)
Inactive	1 Reference	1 Reference
Low	0.75 (0.57, 0.98)	0.93 (0.86, 1.01)
Moderate	0.76 (0.54, 1.06)	0.91 (0.82, 1.01)
High	0.68 (0.46, 1.03)	0.84 (0.73, 0.97)
**Individuals with incident AF**		
Inactive	1 Reference	1 Reference
Low	0.85 (0.68, 1.07)	0.89 (0.83, 0.97)
Moderate	0.97 (0.75, 1.25)	0.88 (0.79, 0.97)
High	0.89 (0.67. 1.19)	0.75 (0.68, 0.84)

All models were adjusted for age, sex, body mass index, education, smoking status, frequency of alcohol consumption and study cohort. AF: Atrial fibrillation, RR: Risk ratio, CI: Confidence interval.

Table S16.

Risk ratio for stroke and mortality after exclusion of individuals reporting coronary heart disease at baseline.

	**Stroke**	**All-cause mortality**
**Total cohort**	RR (95% CI)	RR (95% CI)
Inactive	1 Reference	1 Reference
Low	0.90 (0.82, 0.99)	0.88 (0.84, 0.93)
Moderate	0.78 (0.70, 0.87)	0.82 (0.77, 0.87)
High	0.81 (0.72, 0.91)	0.78 (0.73, 0.84)
**Individuals with AF**		
Inactive	1 Reference	1 Reference
Low	0.84 (0.68, 1.03)	0.90 (0.85, 0.96)
Moderate	0.89 (0.70, 1.13)	0.89 (0.83, 0.97)
High	0.89 (0.68, 1.15)	0.79 (0.71, 0.87)

All models were adjusted for age, sex, body mass index, education, smoking status, frequency of alcohol consumption and study cohort. AF: Atrial fibrillation, RR: Risk ratio, CI: Confidence interval.

Table S17.

Risk ratio and confidence interval for stroke after exclusion of individuals with <2 years of follow-up.

	**Total cohort** RR (95% CI)	**Individuals with AF** RR (95% CI)
Inactive	1 Reference	1 Reference
Low	0.90 (0.82, 0.99)	0.81 (0.67, 0.97)
Moderate	0.80 (0.71, 0.89)	0.89 (0.72, 1.11)
High	0.81 (0.72, 0.92)	0.83 (0.65, 1.07)

Adjusted for age, sex, body mass index, education, smoking status, frequency of alcohol consumption, coronary heart disease and study cohort. RR: Risk ratio, CI: Confidence interval. PA: Physical activity, AF: Atrial fibrillation.

Table S18.

Spearman rank correlation between self-reported PA and accelerometery derived parameters in participants with and without AF in Tromsø7.

	Accelerometer derived PA parameters							
	CPM	Sedentary (<1.5 MET)	Light (1.5 – 2.9 MET)	Moderate (3 – 5.9 MET)	Vigorous (6 – 8.9 MET)	Very vigorous (≥9 MET)	MVPA (≥3 MET)	Steps per/day
No AF								
Spearman's ρ	0.28	-0.08	0.05	0.29	0.29	0.19	0.32	0.34
P-value	*<0.001*	*<0.001*	*<0.001*	*<0.001*	*<0.001*	*<0.001*	*<0.001*	*<0.001*
AF								
Spearman's ρ	0.28	-0.07	0.02	0.33	0.29	0.18	0.34	0.32
P-value	*<0.001*	*0.11*	*0.65*	*<0.001*	*<0.001*	*<0.001*	*<0.001*	*<0.001*

Cut-points for intensity specific categories of accelerometery-derived PA were defined according to triaxial vector magnitude CPM as <150: sedentary behaviour, 150-2689: light, 2690-6166: Moderate, 6167-9641: Vigorous, >9642: Very vigorous[33, 49, 50]. PA: Physical activity, MET: Metabolic equivalent of task, AF: Atrial fibrillation, CPM: Counts per minute, MVPA: Moderate to vigorous physical activity.

Table S19.

Device measured PA in participants with and without AF classified according to self-reported PA

	**Inactive**	**Low**	**Moderate**	**High**
**No AF**	(n = 278)	(n = 2 259)	(n = 1 352)	(n = 1 221)
**Accelerometery derived parameters**				
Achieving PA guidelines n (%)	126 (45.3)	1 459 (64.6)	1 060 (78.4)	1 054 (86.3)
CPM (min/day)	429.4 (237.6)	484.8 (209.7)	553.0 (226.7)	599.9 (235.6)
Light PA (min/day)	386.1 (138.0)	396.7 (119.4)	405.5 (114.4)	404.4 (107.0)
Moderate to vigorous PA (min/day)	17.8 (28.4)	29.1 (31.4)	41.9 (37.3)	49.7 (41.7)
Sedentary time (min/day)	602.7 (142.6)	590.3 (130.2)	579.1 (136.7)	577.7 (130.4)
Steps per day	4 998.0 (3 273.0)	6 016.0 (3 278.0)	7 374.5 (3 783.0)	8 144.0 (4 194.0)
**AF**	(n = 35)	(n = 240)	(n = 113)	(n = 107)
**Accelerometery derived parameters**				
Achieving PA guidelines n (%)	.	100 (41.7)	71 (62.8)	78 (72.9)
CPM (min/day)	389.4 (233.0)	413.7 (206.4)	435.4 (208.3)	523.1 (239.3)
Light PA (min/day)	375.3 (160.6)	371.1 (110.4)	368.3 (117.8)	367.1 (114.4)
Moderate to vigorous PA (min/day)	14.9 (17.5)	15.6 (29.3)	26.8 (33.4)	38.7 (45.9)
Sedentary time (min/day)	609.7 (102.3)	591.8 (133.6)	591.6 (127.1)	585.7 (131.7)
Steps per day	4 048.0 (3 868.0)	4 512.5 (3 365.0)	5 493.0 (3 513.0)	7 313.0 (4 619.0)

Continuous variables are presented as median (Interquartile range) due to the non-normal distribution of the data. Based on a subgroup of 5605 (n=495 with prevalent or incident AF) individuals who attended the second visit of the seventh Tromsø Study[24]. Cut-points for intensity specific categories of accelerometery-derived PA were defined according to triaxial vector magnitude counts per minutes (CPM) as <150 CPM: sedentary behaviour, 150-2689 CPM: light, ≥2689 CPM: Moderate to vigorous[33, 49, 50]. Achieving guidelines by accelerometery was defined as ≥150 minutes of MVPA per week. PA: physical activity, AF: Atrial fibrillation, CPM: Counts per minute.

Table S20.

Sex-specific analyses of stroke and mortality.

	**Stroke**			**All-cause mortality**		
	**Men**	**Women**		**Men**	**Women**	
**Total cohort**	RR (95% CI)	RR (95% CI)	*P*-value PA*Sex	RR (95% CI)	RR (95% CI)	*P*-value PA*Sex
Inactive	1 Reference	1 Reference		1 Reference	1 Reference	
Low	0.92 (0.83, 1.03)	0.92 (0.80, 1.05)	*0.824*	0.93 (0.88, 0.98)	0.84 (0.78, 0.90)	*0.021*
Moderate	0.81 (0.71, 0.93)	0.83 (0.70, 0.97)	*0.948*	0.86 (0.80, 0.92)	0.76 (0.70, 0.83)	*0.073*
High	0.83 (0.73, 0.95)	0.85 (0.71, 1.03)	*0.933*	0.82 (0.77, 0.88)	0.71 (0.64, 0.79)	*0.036*
**Individuals with AF**						
Inactive	1 Reference	1 Reference		1 Reference	1 Reference	
Low	0.79 (0.63, 0.99)	0.82 (0.62, 1.08)	*0.980*	0.93 (0.87, 0.99)	0.89 (0.82, 0.96)	*0.622*
Moderate	0.76 (0.58, 0.99)	1.10 (0.80, 1.50)	*0.102*	0.87 (0.80, 0.95)	0.89 (0.81, 0.99)	*0.514*
High	0.80 (0.61, 1.06)	0.81 (0.51, 1.26)	*0.672*	0.82 (0.75, 0.90)	0.71 (0.59, 0.85)	*0.259*

Adjusted for age, body mass index, education, smoking status, frequency of alcohol consumption, coronary heart disease and study cohort. RR: Risk ratio, CI: Confidence interval. PA: Physical activity, AF: Atrial fibrillation.

Table S21.

Interaction analysis between age and physical activity for the outcome all-cause mortality in individuals with atrial fibrillation.

	Age 50<br>RR (95% CI)	Age 60<br>RR (95% CI)	Age 70<br>RR (95% CI)	Age 80<br>RR (95% CI)
Inactive	1 Reference	1 Reference	1 Reference	1 Reference
Low	0.75 (0.55, 1.01)	0.80 (0.67, 0.96)	0.88 (0.82, 0.94)	0.96 (0.93, 0.99)
Moderate	0.56 (0.38, 0.82)	0.67 (0.53, 0.84)	0.81 (0.74, 0.89)	0.95 (0.91, 1.00)
High	0.34 (0.21, 0.55)	0.48 (0.37, 0.64)	0.69 (0.62, 0.78)	0.93 (0.87, 0.98)

A flexible parametric survival model (adjusted for sex, body mass index, education, smoking, frequency of alcohol consumption, coronary heart disease and study cohort) that included an interaction term between PA and age was fit to the data. In this interaction model, the HR for the interaction between age and high PA was larger than 1.00 and statistically significant (HR: 1.028; $P = 0.006$), suggesting that the association between high PA and mortality was attenuated when age increased. Similar observations were made for moderate (HR: 1.015; $P = 0.078$) and low PA (HR: 1.005; $P = 0.449$), although the HR for the interaction term was smaller in magnitude and not statistically significant. Regression standardisation conditional on age (50, 60, 70 and 80 years) was subsequently performed to estimate the age dependent RR associated with PA. PA: Physical activity, HR: Hazard ratio, RR: Risk ratio, CI: Confidence interval, AF: Atrial fibrillation.

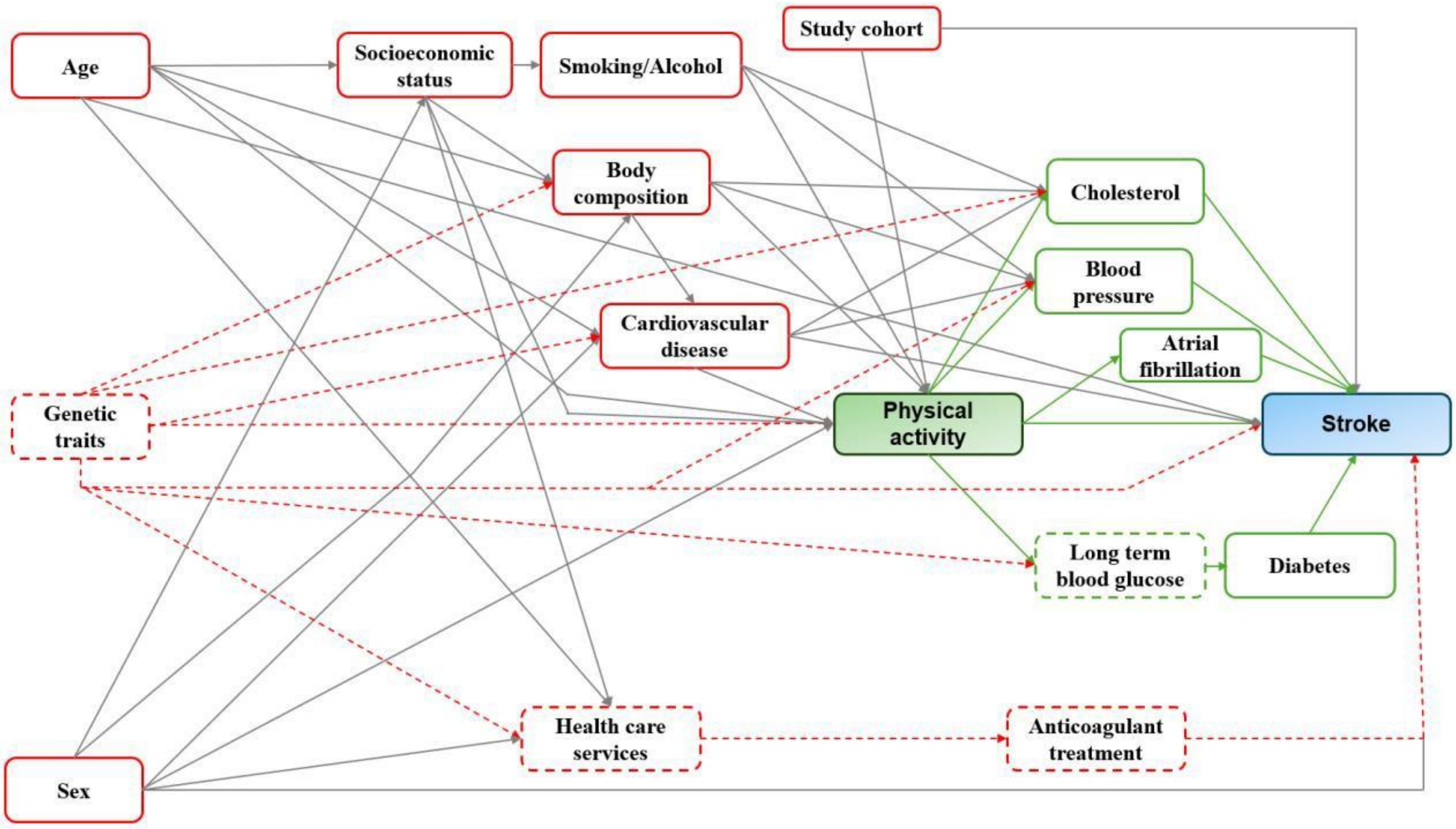

Figure S1.
Simplified directed acyclic graph depicting the relationship between physical activity and stroke. The green node illustrates the exposure of interest while the blue node indicates the primary outcome of interest. Nodes with dashed lines indicate unobserved variables. Ancestors of both the exposure and the outcome (confounders) are illustrated as nodes with red borders. Nodes that are descendants of the exposure and ancestors of the outcome (mediators) are illustrated with green borders. The green arcs depict causal pathways, red arcs depict confounding pathways, and the grey arcs depict blocked pathways. Socioeconomic status was proxied by university education (yes/no). Thus, the association between physical activity level and the outcomes for interest was conditional on age, sex, body mass index, education, smoking status, frequency of alcohol consumption, coronary heart disease and study cohort.

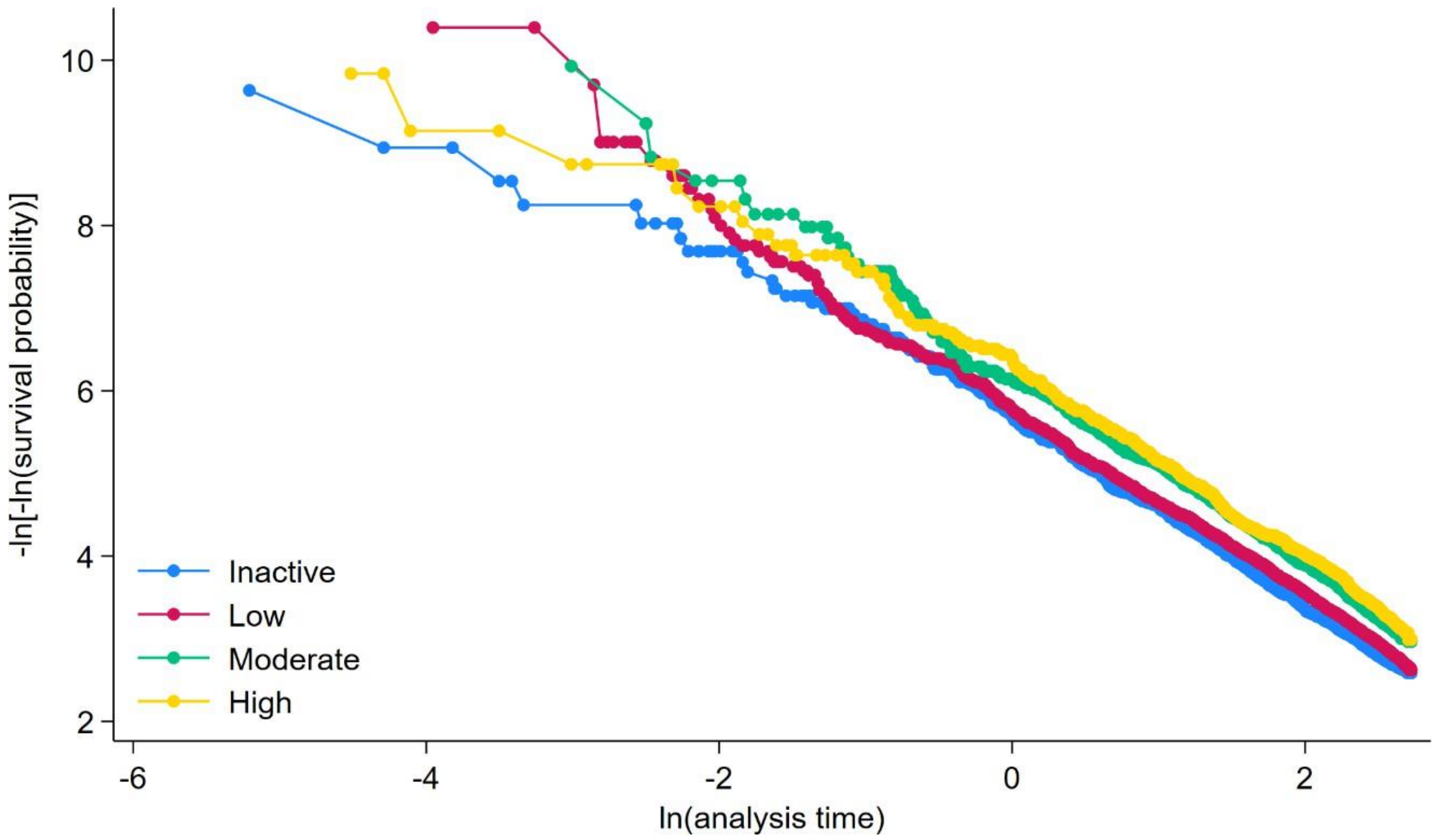

Figure S2.
Log-log plot for the outcome stroke by level of physical activity.

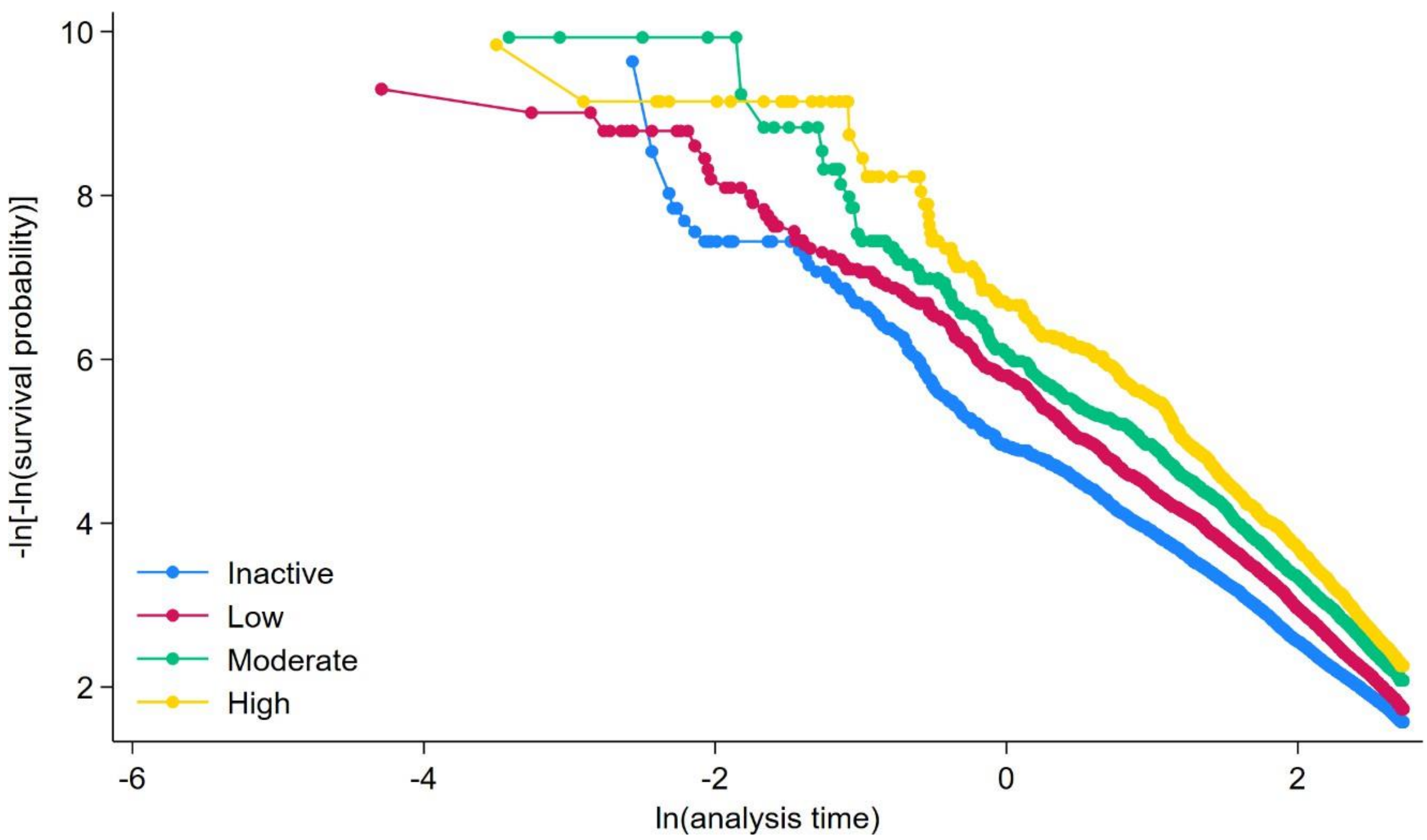

Figure S3.
Log-log plot for the outcome all-cause mortality by levels of physical activity.
